# Hypoxia-inducible factor 1α promotes primary tumor growth and tumor-initiating cell activity in breast cancer

**DOI:** 10.1186/bcr3087

**Published:** 2012-01-07

**Authors:** Luciana P Schwab, Danielle L Peacock, Debeshi Majumdar, Jesse F Ingels, Laura C Jensen, Keisha D Smith, Richard C Cushing, Tiffany N Seagroves

**Affiliations:** 1 Center for Cancer Research and the Department of Pathology and Laboratory Medicine, The University of Tennessee Health Science Center, Cancer Research Building, 19 South Manassas Street, Memphis, TN 38163, USA

## Abstract

**Introduction:**

Overexpression of the oxygen-responsive transcription factor hypoxia-inducible factor 1α (HIF-1α) correlates with poor prognosis in breast cancer patients. The mouse mammary tumor virus polyoma virus middle T (MMTV-PyMT) mouse is a widely utilized preclinical mouse model that resembles human luminal breast cancer and is highly metastatic. Prior studies in which the PyMT model was used demonstrated that HIF-1α is essential to promoting carcinoma onset and lung metastasis, although no differences in primary tumor end point size were observed. Using a refined model system, we investigated whether HIF-1α is directly implicated in the regulation of tumor-initiating cells (TICs) in breast cancer.

**Methods:**

Mammary tumor epithelial cells were created from MMTV-PyMT mice harboring conditional alleles of *Hif1a*, followed by transduction *ex vivo *with either adenovirus β-galactosidase or adenovirus Cre to generate wild-type (WT) and HIF-1α-null (KO) cells, respectively. The impact of HIF-1α deletion on tumor-initiating potential was investigated using tumorsphere assays, limiting dilution transplantation and gene expression analysis.

**Results:**

Efficient deletion of HIF-1α reduced primary tumor growth and suppressed lung metastases, prolonging survival. Loss of HIF-1α led to reduced expression of markers of the basal lineage (K5/K14) in cells and tumors and of multiple genes involved in the epithelial-to-mesenchymal transition. HIF-1α also enhanced tumorsphere formation at normoxia and hypoxia. Decreased expression of several genes in the Notch pathway as well as *Vegf *and *Prominin-1 *(CD133)was observed in response to *Hif1a *deletion. Immunohistochemistry confirmed that CD133 expression was reduced in KO cells and in tumorspheres. Tumorsphere formation was enhanced in CD133^hi ^versus CD133^neg ^cells sorted from PyMT tumors. Limiting dilution transplantation of WT and KO tumor cells into immunocompetent recipients revealed > 30-fold enrichment of TICs in WT cells.

**Conclusion:**

These results demonstrate that HIF-1α plays a key role in promoting primary mammary tumor growth and metastasis, in part through regulation of TICs. HIF-1α regulates expression of several members of the Notch pathway, CD133 and markers of the basal lineage in mammary tumors. Our results suggest that CD133, which has not been profiled extensively in breast cancer, may be a useful marker of TICs in the PyMT mouse model. These data reveal for the first time that HIF-1α directly regulates breast TIC activity *in vivo*.

## Introduction

A hallmark of most solid tumors, hypoxic regions are associated with resistance to radiation and chemotherapy [1,2]. O_2 _tension in advanced breast cancers can be as low as 0.1% to 1% O_2 _[3], a range commonly used to model tissue hypoxia *in vitro*. The oxygen-responsive hypoxia-inducible factor 1α (HIF-1α) protein, a master regulator of the hypoxic response [4], is overexpressed in a variety of carcinomas and their metastases, including breast cancer [5]. A majority of ductal carcinoma *in situ *and almost all poorly differentiated, invasive breast carcinomas overexpress HIF-1α [6]. Moreover, HIF-1α promotes multiple steps of the metastasis program [7]. Either the overexpression of HIF-1α protein or the enrichment of a hypoxic gene signature in the primary tumor correlates with poor prognosis and decreased survival in breast cancer patients [8,9].

Breast cancer cells that exhibit properties of cancer stem cells (CSCs), also referred to as tumor-initiating cells (TICs), are more resistant than bulk tumor cells to therapeutic intervention, including radiation and DNA-damaging drugs [10-12]. Hypoxic culture promotes self-renewal of several cell types, including neurospheres, hematopoietic stem cells (HSCs) and embryonic stem (ES) cells [13]. HIF-1α is also necessary *in vivo *for HSCs because its deletion causes stem cell exhaustion [14].

Accumulating evidence supports the hypothesis that stem cells and TICs exist in a hypoxic niche microenvironment [13]. The direct relationship of the hypoxic response to TIC activity has been demonstrated in adult glioma, human acute myeloid leukemia (AML) and murine lymphoma [15,16]. In gliomas, expression of *HIF2A *mRNA was enriched, and knockdown of *HIF2A*, but not *HIF1A*, reduced TIC activity in patient xenografts [15]. In contrast, HIF-1α was found to be essential for maintaining TIC activity in a syngeneic rodent transplant model of lymphoma and in AML patient xenografts through regulation of the Notch pathway [16]. Interestingly, in gliomas, the TIC population was enriched via cell sorting based on the expression of a single cell surface marker, CD133 (PROM1). PROM1, a transmembrane protein without a known ligand, is a hypoxia-responsive protein regulated by HIF-1α [17,18].

Several studies have shown that the population of breast tumor cells with the ability to self-renew is enriched with the ability to initiate tumorigenesis *in vivo *[19-22]. Furthermore, TICs may drive metastasis [23,24]. The mouse mammary tumor virus polyoma virus middle T (MMTV-PyMT) mouse model is one of the most commonly utilized preclinical mouse models in breast cancer research because tumor latency is short and there is a high frequency of metastasis to the lung [25]. Whole-genome array profiling indicates that PyMT tumors most closely resemble the luminal B subtype of human breast cancer [26], although end-stage PyMT tumors are estrogen and progesterone receptor (ER and PR)-negative [25].

Moreover, the specific contribution of HIF-1α in regulating TICs in breast cancer remains undefined, particularly in the context of syngeneic rodent models that recapitulate the breast cancer microenvironment. It has previously been shown that conditional deletion of *Hif1a *in PyMT+ tumors by crosses to mice expressing MMTV-Cre delayed the onset of palpable tumors, delayed the progression of hyperplasias to carcinomas and reduced lung metastases [27]. We have now created an improved model system in which *Hif1a *is efficiently deleted in the mammary tumor epithelium via *ex vivo *viral transduction with Cre recombinase prior to the injection of mammary tumor cells to the cleared fat pads of recipient mice. Validating the use of a transplantation paradigm in lieu of intact transgenic mice, previous studies have shown that when isolated PyMT tumor cells are serially regenerated as tumors in recipient mice the morphology and gene expression profiles are similar to the tumors found in the transgenic mouse [28].

Using these novel HIF-1α wild-type (WT) and HIF-1α-null (KO) mammary tumor epithelial cells (MTECs), we demonstrate that HIF-1α prominently augments primary tumor growth and lung metastasis and accelerates relapse due to metastasis. Furthermore, we show for the first time that HIF-1α promotes mammary tumorsphere formation and enhances TIC frequency *in vivo*, in part by regulation of the expression of markers associated with the basal lineage, the Notch pathway and CD133. Together these data indicate that suppressing the hypoxic response may be beneficial not only by reducing primary tumor mass but also by suppressing the breast TIC subpopulation that may ultimately be responsible for patient relapse.

## Materials and methods

### Animals

Mice harboring two alleles of exon 2 of *Hif1a *flanked by *loxP *sites (double-*floxed, DF*) were provided by Dr Randall Johnson (University of California San Diego) on a mixed genetic background (129Sv-C57BL/6 [29]). *Hif1a *stock mice were first backcrossed to the FVB/Nj strain (The Jackson Laboratory, Bar Harbor, ME, USA) for 11 generations prior to being bred to MMTV-PyMT transgenic mice obtained from Dr Kent Hunter (National Cancer Institute, Frederick, MD, USA), which had previously been backcrossed to the FVB/Nj strain. Lung metastasis induced by the PyMT transgene is highly penetrant in the FVB/Nj background [30]. All procedures were approved by Institutional Animal Care and Use Committee at the University of Tennessee Health Science Center.

### Establishing *Hif1a *wild-type and knockout mammary tumor epithelial cells

Several mammary tumors (> 500 mm^3^) were isolated from *Hif1a DF*, PyMT+ bigenic female mice. Tumors were chopped with scalpels and then with razor blades, and the paste was digested with 1 mg/ml collagenase type III (Worthington Biochemical Corp, Lakewood, NJ, USA) in RPMI media containing 5% fetal bovine serum (FBS) (5 ml/g tissue) for 2 hours at 37°C. Organoids were pelleted at 1,100 rpm, washed four times with digestion buffer and then plated into standard tissue culture plates in plating medium as described previously [31]. After 48 to 72 hours, the plating medium was switched to complete mammary epithelial cell growth medium (GIBCO DMEM: Nutrient Mixture F-12 (DMEM/F-12; Invitrogen, Carlsbad, CA, USA), 5% FBS, 5 μg/ml insulin (Sigma-Aldrich, St Louis, MO, USA), 10 ng/ml recombinant murine epidermal growth factor (EGF) (Invitrogen)). At passage 6, MTECs were transduced with either adenovirus β-galactosidase (adeno-β-gal) or adeno-Cre at a multiplicity of infection (moi) of 80 plaque-forming units (pfu)/cell to generate WT and KO MTECs, respectively. Adenoviral transduction was repeated, and the deletion efficiency between WT and KO MTECs was confirmed by both quantitative RT-PCR (qRT-PCR) and Western blot analysis using the reagents presented in Additional file 1 Tables S1 and S2. After adenoviral transduction, MTECs were weaned to medium containing only 2% FBS (DMEM/F-12 + 2% FBS).

For subcultivation, cells were rinsed twice with Puck's A saline, then incubated for up to 60 minutes at 37°C in a 3:1 solution of dispase II/0.25% trypsin reconstituted in Puck's A. No ethylenediaminetetraacetic acid (EDTA) was utilized to subcultivate cells because treating cells with trypsin-EDTA changed tumor cell morphology from an epithelial (cuboidal) to a mesenchymal-like (spindle) appearance. All cells were passaged less than 30 times before use in tumorsphere or *in vivo *assays. Spent media were routinely tested for mycoplasma using the MycoAlert Kit (Lonza, Basel, Switzerland).

All cells were grown either at normoxia in an air-jacketed CO_2 _incubator (5% CO_2_; SANYO, Wood Dale, IL, USA) or at hypoxia (0.5% O_2_, 5% CO_2_) in a multigas incubator (SANYO) in which N_2 _gas displaces O_2_. Cells were exposed acutely (≤6 hours) or chronically (> 6 hours to several days) to hypoxia and were removed from chronic hypoxic exposure only for brief periods to change the media.

For immunostaining of cultured cells, WT or KO cells passaged with a dispase-trypsin mixture were plated into tissue culture-treated chamber well slides (BD Biosciences, Franklin Lakes, NJ, USA), grown to 60% to 80% confluence and then postfixed for 20 minutes at room temperature with 4% paraformaldehyde (PFA)-PBS, followed by permeabilization with 0.5% Triton X-100 for 5 minutes. Cells stained with CD133 were not permeabilized. Primary antibodies to CD133, Troma-I (K8), cytokeratin 14 (K14) and cytokeratin 5 (K5) were incubated overnight at 4°C using the dilutions listed in Additional file 1 Table S2. All cells were stained with 4',6-diamidino-2-phenylindole (DAPI) prior to being mounted with VECTASHIELD Mounting Medium (Vector Laboratories, Burlingame, CA, USA).

### Western blot analysis

Insoluble material remaining after preparation of whole-cell extract (WCE) was reextracted in high-salt (HS) solution (400 mM NaCl) buffer as described previously [32], except that the deubiquitinase inhibitor *N*-ethylmaleimide (NEM) was added to a final concentration of 0.5 μM. HS-WCE was resolved on 3% to 8% Tris-acetate gels (1 to 10 μg/lane; Invitrogen) and transferred onto polyvinylidene fluoride membranes prior to blocking with 5% milk and enhanced chemiluminescence-based detection of antibody complexes. All antibodies and dilution factors are listed in Additional file 1 Table S2.

### Gene expression

Total RNA was prepared using RNA-Bee RNA isolation reagent (amsbio, Lake Forest, CA, USA), and RNA quality was confirmed by the Agilent 2100 Bioanalyzer (Agilent Technologies, Santa Clara, CA, USA) assay. RNA with an RNA integrity number > 9.0 was used to prepare cDNA using the High-Capacity cDNA Reverse Transcription Kit (Applied Biosystems, Foster City, CA, USA). qRT-PCR was performed using optimized primer and 6-carboxyfluorescein-labeled probe sets designed using Universal ProbeLibrary Assay Design Center software (Roche Applied Science, Indianapolis, IN, USA) as described previously [33]. To control for cDNA input (40 to 80 ng/reaction) when using cultured cells as the source of RNA, the crossing point (*C*_p_) values were normalized based on the expression of the integrator complex subunit 3 (*Ints3*) gene, which is expressed at moderate levels in the mammary gland. This gene changes < 20% between WT and KO cells cultured at normoxia or hypoxia and in WT and KO tumors by Illumina whole-genome expression arrays (Illumina Inc, San Diego, CA, USA) (TNS, personal observations). To compensate for any changes in epithelial content in whole tumors between genotypes, because only the tumor epithelium is deleted for *Hif1a, Ints3-*normalized *C*_p _values were also normalized to *Krt18 *(cytokeratin 18).

### Cell growth and invasion assays

MTECs were grown in normoxic culture (ambient air; 5% CO_2_) or hypoxic culture (0.5% O_2_, 5% CO_2_) in medium buffered with 25 mM 4-(2-hydroxyethyl)-1-piperazineethanesulfonic acid (HEPES). On the day before enumeration, either 350,000 cells/well in 6-well format (complete growth medium) or 100,000 cells/well in 12-well format (2% FBS medium) were seeded into multiwell plates. In all cases, the medium was changed postplating, but was not replenished for the duration of the experiment. Cells were harvested after culture for 0, 24, 48, 72 or 96 hours of culture at normoxia and hypoxia. All cells were plated in triplicate or quadruplicate per genotype/oxygen tension/time point. Each replicate was counted by hemacytometer, and counts were verified using an Accuri personal flow cytometer after gating against cell debris (BD Biosciences).

For invasion assays, WT and KO MTECs that had been gradually weaned to medium supplemented with 0.5% FBS were cultured overnight in serum-free DMEM/F-12 medium. The next day 25,000 cells were plated onto control inserts or Matrigel-coated transwell inserts (BD Biosciences) and attractedto wells containing complete growth medium with 5% FBS. Cells were plated in triplicate per genotype/oxygen tension. The mean cell invasion index corrected for random migration was calculated after 48 hours according to the manufacturer's instructions. Changes in invasion are expressed as a fold changes relative to the invasion index observed for WT cells cultured at normoxia (fold change = 1.0).

### Mammary tumor epithelial cell transplant into FVB recipients

MTECs dissociated into single cells with 0.05% trypsin-EDTA were counted using a hemacytometer and diluted into HBSS. When transplanted into recipients at relatively low density (≤500 cells/gland), cells were diluted 1:1 (vol:vol) with growth-factor reduced Matrigel-Hank's balanced salt solution (HBSS). At higher densities, cells were resuspended in HBSS alone. Cells were kept on ice until injection into the right inguinal mammary fat pads (10 μl) of 3-week old female FVB/Nj recipients (The Jackson Laboratory) using a 26-gauge PT2 needle mounted on a Hamilton syringe, followed by clearing of the endogenous epithelium. Recipients were palpated one or two times per week, and outgrowths were measured with digital calipers to calculate tumor volume as described previously [27].

### Tissue histology and immunostaining

Tumors were harvested from anesthetized mice and flash-frozen in liquid nitrogen for preparation of RNA or protein, or they were fixed in 10% neutral buffered formalin (NBF) for 6 hours at room temperature for histological staining (H & E) and immunostaining. Paraffin-embedded sections (5 to 7 μm) were immunostained after antigen retrieval (1 × citrate buffer) using the primary antibodies listed in Additional file 1 Table S2, followed by development with a VECTASTAIN Elite ABC Kit and ImmPACT DAB (diaminobenzidine) substrate (Vector Laboratories). Alternatively, to prepare frozen sections suitable for CD133, Troma I, K14 and K5 immunofluorescent staining, anesthetized mice were perfused intracardially with 10% NBF. Tumor tissue was postfixed for 10 minutes in NBF at room temperature prior to cryoprotection overnight at 4°C in 30% sucrose-PBS, embedding in OCT medium and preparation of 10 μm sections by cryostat. Sections used for visualizing the keratins were postfixed for an additional 10 minutes in 10% NBF prior to 0.5% Triton X-100 permeabilization. All antibodies and staining conditions are listed in Additional file 1 Table S2.

### Lung metastasis and survival studies

Lungs were either harvested from recipients at the same time as primary tumors (at a volume of approximately 1,000 mm^3^), 8 weeks after primary tumor resection (500 to 750 mm^3^) or when recipients subjected to tumor resection were moribund as indicated by body weight loss > 15% and panting. Lungs were inflated with 10% NBF and postfixed in NBF overnight. Paraffin-embedded sections (7 μm) representing every 100 μm of lung tissue were obtained for each paraffin block, and all sections were stained with H & E. The individual H & E-stained section containing the highest number of metastases per recipient, as evaluated by counting each slide under a light microscope at × 50 original magnification, was used to determine the grand mean of metastases per genotype.

### Tumorsphere culture and immunostaining

WT or KO MTECs were briefly trypsinized, washed and strained (40 μm filter) to obtain single cells. The presence and viability of single cells were verified by viewing trypan blue-stained cells using a hemacytometer, and only cells with > 90% viability were used in sphere assays. Single cells were resuspended in serum-free DMEM-F-12 mammosphere media containing 20 ng/ml mouse recombinant EGF, 20 ng/ml basic fibroblast growth factor, 1 × B27 (all from Invitrogen) and 4 μg/ml heparin (Sigma-Aldrich, St Louis, MO, USA) as described previously [34]. Primary tumorspheres were derived by plating 30,000 single cells/well into six-well ultra-low-adhesion dishes. Secondary and tertiary tumorspheres were plated at 5,000 to 10,000 cells/well and 2,000 cells/well, respectively. Dishes were cultivated at normoxia or hypoxia (0.5% O_2_; 5% CO_2_) for 10 to 14 days prior to enumeration of spheres. Individual spheres ≥ 100 μm from each replicate well (*n *≥ 12 wells/genotype/oxygen tension) were counted under a dissecting microscope. The percentage of cells capable of forming spheres, termed the "sphere formation efficiency" (SFE), was calculated as follows: [(number of spheres formed/number of single cells plated) × 100]. End point tumorspheres were collected from ultra-low-adhesion dishes, washed with PBS, then flash-frozen for preparation of RNA or dried onto slides for 10 to 15 minutes at 37°C. Slides were postfixed with 4% PFA-PBS for 15 minutes and immunostained with the antibodies indicated in Additional file 1 Table S2. All slides were stained with DAPI prior to being mounted in either ProLong Gold antifade reagent (Invitrogen) or VECTASHIELD Mounting Medium (Vector Laboratories).

### Tumor digestion and flow sorting

All mammary gland tumors used for flow-sorting experiments were harvested from intact *Hif1a DF*, MMTV-PyMT+ (equivalent to HIF-1α WT) transgenic female mice at a size of > 300 mm^3 ^but < 1,500 mm^3^. Necrotic areas were removed from solid tumor tissue, and solid tissue was cut into small fragments with scissors. Tumor tissue was then weighed and chopped for 5 minutes with scalpels, followed by 5 minutes of chopping to a fine paste with razor blades. The tissue paste was digested in digestion buffer (DMEM/F-12, 1 × gentamicin, 1 × antibiotic-antimycotic (Sigma-Aldrich), 300 U/ml collagenase type III (Worthington Biochemical Corp) and 100 U/ml hyaluronidase (Sigma-Aldrich)) for 1 to 2 hours at 37°C at 125 rpm (10 ml/g tissue). Digested tissue was pelleted by centrifugation, and the red blood cells were lysed with a solution of 0.8% NH_4_Cl-HBSS. To obtain single cells, organoids were further digested with 0.25% trypsin-EDTA for up to 10 minutes at 37°C and washed with serum-containing medium to inactivate trypsin, followed by a final digestion for 10 minutes at 37°C in 5 mg/ml dispase II (Roche Applied Science)/Puck's A solution containing 1 mg/ml DNase I (Roche Applied Science). The cell suspension was passed through a 40 μm filter insert, and this flow-through was then passed through a flow cytometer tube with a 35 μm filter cap insert to further enrich for single cells. Viability postdigestion was routinely assessed by trypan blue staining. Only cells with viability > 85% were subjected to antibody staining.

Isolated single cells were resuspended to a density of up to 5 million cells/ml in flow buffer (HBSS, 2% FBS, 10 mM HEPES) and incubated for 1 hour on ice with the biotin- or fluorophore-conjugated antibodies listed in Additional file 1 Table S2. These antibodies included a biotin-conjugated anti-mouse hematopoietic lineage panel supplemented with anti-mouse CD31-biotin, anti-mouse CD133-phycoerythrin (CD-133-PE) and anti-mouse CD24-fluorescein isothiocyanate (CD24-FITC), each at a 1:100 dilution. Secondary antibody incubation (streptavidin-allophycocyanin) in flow buffer was performed for 30 minutes on ice. After being stained, cells were strained through a clean flow cytometer tube with a 35 μm filter cap. To determine cell viability, either SYTOX Blue (1 μM; Invitrogen) or 7-AAD (1 μg/ml; BD Biosciences) was added to each flow tube sample 10 to 20 minutes before flow cytometry analysis.

Cells were subjected to cytometric profiling using a 100-μm nozzle at a sheath pressure of 20 psi on a BD Biosciences FACSAria flow cytometer (maintained by the University of Tennessee Health Science Center Flow Cytometry and Sorting Core) equipped with violet (404 nm), blue (488 nm), green (532 nm) and red (635 nm) lasers and applying FACSDiva software. All sample and collection tubes were maintained on ice. BD Biosciences CompBeads stained with either CD133-PE or CD24-FITC were used to set the compensation gates, and cells stained with isotype-matched secondary antibodies were used as controls for staining specificity. Cells heat-killed for 30 minutes at 65°C were used to gate against dead cells positive for SYTOX Blue or 7-AAD. Lineage-negative (Lin^neg^) cells were identified by gating against cells positive for the mouse lineage panel + CD31. Because almost 100% of cells isolated from late-stage carcinomas of the PyMT-transgenic mouse were positive for CD24-FITC as previously described [19], viable, Lin^neg ^mammary tumor cells were gated in a two-way sort for CD133^hi ^versus CD133^neg ^cells.

Sorted cell samples were collected directly on ice into 15 ml conical tubes precoated with 20% FBS, and a subset of collected cells was subjected to postsorting analysis to verify the purity and viability of the sorted populations. Sorted cell populations were immediately pelleted, washed to remove FBS and counted using a hemacytometer prior to reconstitution in mammosphere medium and plated in six-well ultra-low-adhesion dishes (Corning Life Sciences, Corning, NY, USA) at the indicated densities. FACSDiva plots were imported into FlowJo version 7.0 software (Tree Star Inc, Ashland, OR, USA) for data analysis and creation of histogram plots.

### Limiting dilution transplantation

WT or KO MTECs were briefly trypsinized, washed with HBSS and serially diluted into 1:1 HBSS/Matrigel. Six dilutions per genotype (500, 200, 100, 50, 25 or 10 cells/10 μl) were introduced into the right inguinal cleared fat pads of FVB/Nj recipients. Tumor-initiating potential was defined as the ability to form a palpable tumor mass > 5 mm diameter (approximately pea-sized). The estimated TIC frequency was calculated using Extreme Limiting Dilution Analysis (ELDA) software [35]. Fisher's exact (χ^2^) test was used as a complementary approach to compare TIC activity between WT and KO cells at each cell density evaluated (GraphPad Prism version 4.0 software; GraphPad Software, Inc, San Diego, CA, USA). All animals were palpated twice weekly until surgical resection of the primary tumor or euthanasia.

### Image acquisition

Western blots were scanned at 600 dpi and imported into Photoshop software (Adobe Systems, Inc, San Jose, CA, USA). Digital images of immunostained or H & E-stained tissue sections and cells cultured on chamber well slides were captured using a Leica DM6000 upright fluorescence microscope (Leica Microsystems, Inc, Buffalo Grove, IL, USA) mounted with a SPOT Insight cooled charge-coupled device camera (SPOT Imaging Solutions, Inc, Sterling Heights, MI, USA) and imported into Photoshop using SimplePCI software (Hamamatsu Photonics, Sewickley, PA, USA). All images between genotypes and conditions per experiment were digitally captured for the same exposure time. Immunostaining images were not digitally altered to reduce background or to adjust brightness or gain. Confocal images of spheres were captured every 0.75 μm with a Zeiss LSM210 microscope system (Carl Zeiss, Thornwood, NY, USA). Appropriate gain and black level settings were determined based on spheres incubated with secondary antibody alone. All images were captured using the same gain and exposure settings, and upper and lower thresholds were set using the range indicator function. No additional background correction algorithms were applied. Digital slices were merged into a single, composite image using Zeiss Zen software.

## Results

### A mammary tumor model with constitutively deleted *Hif1a*

We observed that HIF-1α expression generally increased during tumor progression in MMTV-PyMT mice as previously observed in patients [6], thereby confirming that the MMTV-PyMT model is appropriate for these studies (Additional file 2 Figure S1). To generate a parental pool of MTECs, mammary tumors were isolated from *Hif1a floxed *(*DF*), PyMT+ females (FVB/Nj background), and epithelial cells were transduced with ether adeno-β-gal or adeno-Cre to create HIF-1α WT and KO cell lines, respectively. Efficient *Hif1a *deletion was confirmed by qRT-PCR and Western blot analysis (Additional file 2 Figure S1).

**Figure 1:**
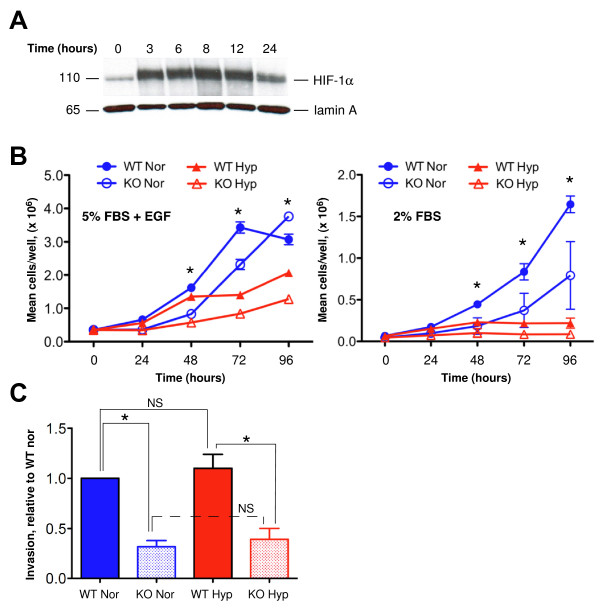
**Effect of *Hif1a *deletion upon growth and invasion**. **(A) **Wild-type (WT) mammary tumor epithelial cells (MTECs) grown to 80% confluence were subjected to hypoxic culture for the indicated times up to 24 hours, or cells continued to be cultured under normoxic conditions such that the time *t *= 0 sample was harvested on the same day as the time *t *= 24 hours hypoxic condition sample. High-salt enriched whole-cell extracts were resolved on 3% to 8% Tris-acetate gels and blotted onto polyvinylidene fluoride membrane, which was divided horizontally at approximately 60 kDa. The top half of the blot was used to detect HIF-1α, and the lower portion was used to detect lamin (loading control) to avoid the need to strip and reprobe the blot. **(B) **Growth curve of WT and knockout (KO) MTECs cultured at normoxia (Nor) or hypoxia (Hyp) in growth medium supplemented with 5% fetal bovine serum (FBS) + epidermal growth factor (EGF) (left) or with 2% FBS (right). For cells grown in 5% FBS + EGF, a representative graph is shown in which the mean ± SEM of cell number per time point of quadruplicate wells per genotype/oxygen tension is plotted per time point. For cells grown in 2% FBS, the grand mean ± SEM of cell number is presented, which was calculated as an average of the mean cell number observed per replicates per time point as observed in three replicate experiments. All data were analyzed by two-way analysis of variance (ANOVA, *P < 0.05). **(C) **The mean fold change (FC) in invasion was normalized to the invasion index observed for WT cells cultured at normoxia (FC = 1.0). Data represent the mean FC in invasion observed in three independent experiments. All columns were compared using one-way ANOVA with a Bonferroni posttest. *P < 0.05.

During expansion, MTECs were weaned from complete growth medium to medium supplemented only with 2% FBS, as low-serum medium was previously utilized to evaluate cell growth [27]. The levels of HIF-1α detected by Western blot analysis following acute (0 to 6 hours) or prolonged (> 6 hours) hypoxia was determined using WT MTECs. On the initial day of hypoxia exposure, cells were 80% confluent prior to transfer to a hypoxic chamber for 24, 12, 8, 6 and 3 hours prior to harvest. Cells taken from normoxic culture (labelled as time *t *= 0) were harvested at the same time as cells that had been exposed to hypoxic conditions for 24 hours. Similar to the profile observed in Hep3B cells [36], HIF-1α peaked by 3 hours of exposure to 0.5% O_2_, and, by 24 hours, expression had attenuated to basal levels (Figure 1A). This decrease in HIF-1α expression may be due HIF-1α's role in transcriptional regulation of the prolyl hydroxylase enzymes, which mediate HIF-1α's turnover by the proteasome, thereby creating a negative feedback loop [37].

In contrast, when WT cells were cultured in the presence of serum and EGF, HIF-1α levels were not attenuated by prolonged hypoxia exposure (Additional file 2 Figure S2), and, in fact, HIF-1α stabilization at hypoxia remained robust for at least 24 hours. Compared to the levels of HIF-1α expression observed in cells cultured in 2% FBS alone, EGF treatment increased HIF-1α levels approximately fourfold at normoxia (time *t *= 0), and this upregulation increased to > 10- or > 20-fold following 3 or 24 hours in hypoxic culture, respectively. The effect was not due to the differences in serum or insulin concentrations between culture media because WT cells cultured in 5% FBS + 5 μg/ml insulin without EGF expressed levels of HIF-1α similar to those of cells cultured in 2% FBS alone (data not shown). Overall, these results indicate that EGF is a potent regulator of HIF-1α expression in normoxic PyMT mammary tumor cells, as previously observed in SK-BR-3 and MCF-7 cells [38], and that EGF prolongs HIF-1α stabilization under hypoxic conditions.

### HIF-1α promotes cell growth and invasion

To determine whether the HIF-1α KO PyMT+ MTECs derived from *ex vivo *adenoviral transduction behave similarly *in vitro *as previously described [27], cell growth was compared in WT and KO cells cultured at normoxia and hypoxia. Cell growth was also compared for WT and KO cells cultured in complete or minimal (2% FBS only) growth medium. By 48 hours of culture, and then throughout the time course, there was a statistically significant difference between WT and KO cell number at normoxia and hypoxia for each medium formulation (Figure 1B); overall, fewer KO than WT cells were observed following exposure to either oxygen tension. Cells cultured in complete growth medium grew faster than those in reduced serum. In cells grown in 5% FBS + EGF, more KO than WT cells were often observed by 96 hours of culture at normoxia (Figure 1B), owing to the acidic environment of the superconfluent WT cells as reported previously [39]. As expected, hypoxia exposure decreased the rate of growth for both WT and KO cells, although the difference between WT and KO cell density was the most striking following extended hypoxia exposure (≥ 72 hours).

Cells cultured in 2% FBS medium were gradually weaned to reduced serum (0.5% FBS) prior to use in invasion assays since immediate serum withdrawal resulted in massive cell death even when cells had been routinely cultured in medium containing 2% FBS. Deletion of *Hif1a *reduced invasion at normoxia by 3.9-fold and by 3.5-fold at hypoxia (Figure 1C). In contrast, significant induction of invasion by WT cells during hypoxia was not observed as previously described [27]. Although there was a trend toward increased invasion, the differences were not significant on the basis of analysis of variance (ANOVA) analysis. Likewise, there was no significant difference in the invasion potential of KO cells between normoxic and hypoxic culture.

### Expression of basal markers is reduced in cultured knockout cells

**Figure 2:**
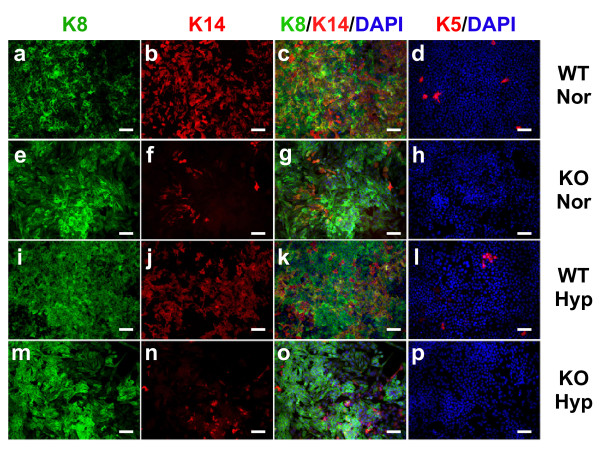
**Loss of basal marker expression in cultured HIF-1 knockout cells**. Wild-type (WT) or knockout (KO) cells were plated onto tissue-culture-treated slides and grown to subconfluence, at which time subsets of cells were exposed overnight to hypoxic (Hyp) culture or remained under normoxic (Nor) culture. Cells were costained with Troma-I (K8, green) and K14 (red) **(a) **to **(c)**, **(e) **to **(g)**, **(i) **to **(k) **and **(m) **to **(o) **or with K5 alone **(d)**, **(h)**, **(l) **and **(p)**; images showing either the green (K8) or red (K14) channel from the co-stained slides are also presented **(a) **to **(b)**, **(e) **to **(f)**, **(i) **to **(j) **and **(m) **to **(n)**. All slides were counterstained with 4',6-diamidino-2-phenylindole (DAPI). Images were captured at ×200 original magnification (scale bar = 50 μm).

The expression of markers of the luminal lineage (Troma-I, detecting cytokeratin 8, K8) and the basal lineage (cytokeratins 5 and 14, K5 and K14) by WT and KO cells was evaluated by immunofluorescent staining of nearly confluent cells that were cultured overnight (12 to 14 hours) at normoxia or hypoxia (Figure 2). The majority of WT and KO MTECs cultured at normoxia (Figures 2a and 2e) and hypoxia (Figures 2i and 2m) stained with antibodies to K8. This was expected because the PyMT model is a luminal-like model of breast cancer. However, fewer KO cells expressed K14 when cultured at either normoxia (Figure 2b vs 2f) or hypoxia (Figure 2j vs 2n). Hypoxic culture downregulated K14 expression in both WT and KO cells (Figure 2b vs 2j and Figure 2f vs 2n). Overall, fewer KO cells were dual-positive for K8 and K14 (yellow cells), suggesting a reduction in a bipotent progenitor cell population in response to HIF-1α deletion. The most striking phenotype was that no K5+ cells could be detected in KO cells cultured at either normoxia (Figure 2d vs 2h) or hypoxia (Figure 2l vs 2p). Hypoxic induction of K5 mRNA has previously been reported in MCF-7 cells, [40]; however, an increase in the number of K5+ cells was not apparent in PyMT WT cells exposed to hypoxia (Figure 2d vs 2l).

### Primary mammary tumor growth is HIF-1α-dependent

**Figure 3:**
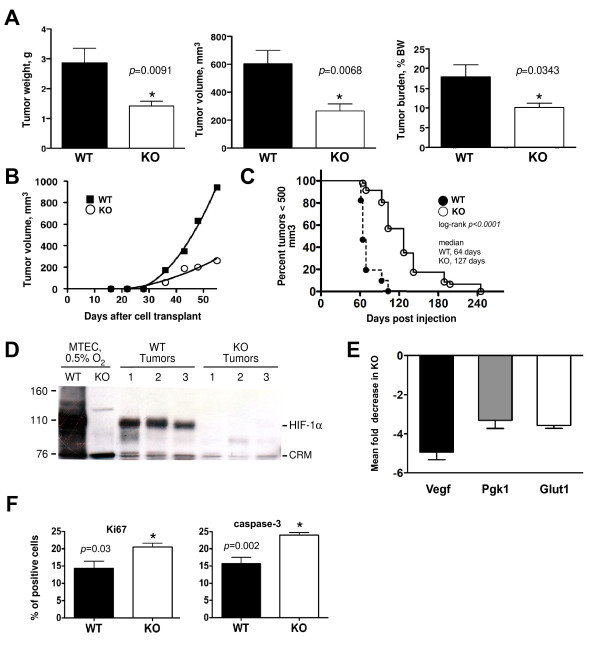
**Deletion of *Hif1α *decreases primary tumor growth**. **(A) **Wild-type (WT) or knockout (KO) cells (*n *= 50,000) were transplanted into FVB/Nj mice. All tumors were harvested at day 56 to evaluate tumor weight, volume and burden (percentage tumor weight/total body weight, BW) (*n *= 10 recipients/genotype, *P *< 0.05, unpaired Student's *t*-test). **(B) **The growth rate of WT and KO tumors following transplant of 50,000 cells. Best-fit curves were established based on a polynomial fit algorithm using GraphPad Prism 4.0 software. Data in (A) and (B) are representative of seven independent experiments (> 60 recipients/genotype). **(C) **When 500 WT and KO cells were implanted into the mammary fat pad, the median time until 50% of recipients developed tumors > 500 mm^3 ^was 64 days for WT mice and 127 days for KO mice (*n *= 14 recipients/genotype, *P *< 0.001, logrank test). **(D) **Western blot for HIF-1α in three independent tumors (500 to 750 mm^3^) per genotype. WT and KO cells that were cultured for 6 hours under hypoxic conditions served as positive and negative controls, respectively. CRM, cross-reactive material. MTEC, mammary tumor epithelial cell. **(E) **Mean fold change ± SEM in expression of HIF-1 targets in KO tumors as determined by qRT-PCR (*n *= 5 tumors/genotype). Decreased gene expression in KO tumors is presented as a negative fold-change relative to WT tumors. **(F) **An increase in Ki67+ cells in KO tumors is balanced by an increase in caspase 3-positive cells (*n *= 5 tumors/genotype, *P < 0.05, Student's *t*-test). Representative immunostaining images are shown in Additional file 2 Figure S4.

To evaluate the contribution of HIF-1α to primary tumor growth in a syngeneic transplant approach, WT or KO MTECs (*n *= 50,000) were injected into single, cleared inguinal mammary fat pads of FVB/Nj recipient females. Single-side injections were utilized to prevent one tumor from influencing the outgrowth of a contralateral tumor. There was an approximately 60% decrease in the wet weight and volume of KO tumors at week 8 posttransplant, when recipients bearing WT tumors required euthanasia in compliance with institutional maximum tumor size recommendations (Figure 3A). Moreover, the rate of KO tumor growth was slower over the entire course of tumor development (Figure 3B). When fewer WT or KO cells were utilized to generate mammary tumors (500 cells/gland), there was a more pronounced delay in the ability of KO cells to form a tumor with a volume > 500 mm^3 ^(Figure 3C). The median time to form large tumors increased from 64 days for WT cells to 127 days for KO cells (Figure 3C).

To confirm that end point KO tumors did not express HIF-1α, Western blotting was performed using extracts isolated from three randomly selected whole WT or KO tumors. Very low levels of HIF-1α were detected in KO end-stage tumors (Figure 3D). Residual expression was likely observed because of the presence of stromal components derived from the host recipients, such as tumor-associated macrophages. As expected, the expression of three classic HIF-1 target genes, vascular endothelial growth factor (*Vegf)*, phosphoglycerate kinase 1 (*Pgk1) *and glucose transporter 1 (*Slc2A1 *or *Glut1*), was reduced in KO tumors (Figure 3E). However, the gross histopathology of tumors was not affected by deletion of *Hif1a*; all tumors were poorly differentiated, solid adenocarcinomas as previously described in the PyMT-transgenic mouse [25] (Additional file 2 Figure S3).

Because WT tumors were larger than KO tumors, they also exhibited more extensive necrosis than KO tumors. High-resolution, digitally scanned whole slides of H & E-stained tumors may be viewed using a link to a public database (refer to the Additional material). Because a larger percentage of KO tumor cells were viable, it is not surprising that more Ki67 and caspase 3-positive cells in KO tumors were detected by immunostaining (Figure 3F and Additional 2, Figure S4). Overall, these changes would be expected to have a zero net effect on KO tumor growth. In addition, as previously reported for late-stage PyMT carcinomas [25], tumors derived from transplanted WT or KO cells did not exhibit any cells positive for ERα by immunostaining (Additional file 2 Figure S5).

### HIF-1α promotes an epithelial-to-mesenchymal transition phenotype and regulates K5 expression *in vivo*

**Figure 4:**
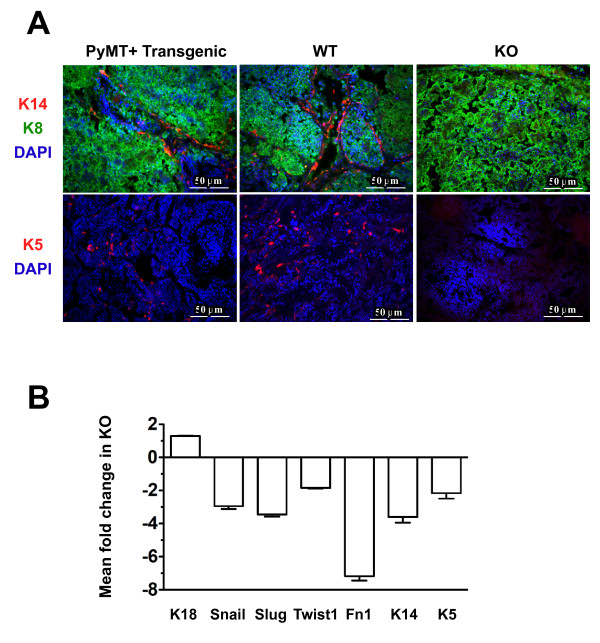
**Loss of cells expressing K14 or K5 in HIF-1α knockout tumors is accompanied by a reduction in the expression of markers of the epithelial-to-mesenchymal transition**. **(A) **Frozen sections were prepared from tumors harvested from PyMT+ transgenic mice or from wild-type (WT) and knockout (KO) tumors harvested from FVB recipients. All tumors were ≥ 500 mm^3 ^in volume. Sections were costained with antibodies to K14 (red) and K8 (green) or K5 (red) alone and counterstained with DAPI. Images were captured at ×200 original magnification. Scale bar = 50 μm. **(B) **The mean fold change ± SEM in gene expression observed in end-stage KO tumors of *Krt14 *(K14) and *Krt5 *(K5) mRNAs, as well as multiple markers of the epithelial-to-mesenchymal transition, as determined by qRT-PCR performed using the primers and probes listed in Additional file 1 Table S2 (*n *= 5 tumors/genotype). The mean ± SEM of biological replicates is graphed. The expression of *Snail, Slug, Twist1, Fn1, Krt14 *(K14) and *Krt5 *(K5) mRNAs was reduced in KO tumors compared to WT tumors; however, the expression of *Krt18 *(K18) varied by < 20% between genotypes.

To determine whether the expression of K5 and K14 was reduced in KO tumors as in cultured cells, expression of K8, K14 and K5 was also evaluated by immunofluorescent staining of end-stage tumors harvested from FVB recipients bearing WT or KO tumors (four tumors/cohort). The immunostaining pattern was compared to that observed in tumors harvested from intact PyMT-transgenic mice. In contrast to results obtained in cultured cells, very few cells were dual-positive for K8 and K14 in WT tumors. Furthermore, no cells positive for K5 or K14 were detected in any KO tumors (Figure 4A) (four tumors/genotype). In contrast, there were no obvious changes in the expression or localization of p63 detected by colorimetric immunostaining of paraffin-embedded sections (Additional file 2 Figure S5; refer also to supplementary database of digitally scanned whole slides).

qRT-PCR analysis of RNA prepared from the same tumors for which sections were prepared for immunostaining revealed that *Krt14 *(K14) and *Krt5 *(K5) mRNA levels were reduced in KO tumors by 3.8- and 2.0-fold, respectively. As expected based on the ability of hypoxia to promote an epithelial-to-mesenchymal transition (EMT) phenotype through the Notch pathway [41], multiple core genes in the EMT signature were downregulated in KO tumors compared to WT tumors, including *Snail, Slug, Twist1 *and *Fn1 *(fibronectin), whereas expression of *Krt18 *(K18) was not affected by HIF-1α deletion (Figure 4B).

### Constitutive *Hif1a *deletion represses metastasis and prolongs survival

**Figure 5:**
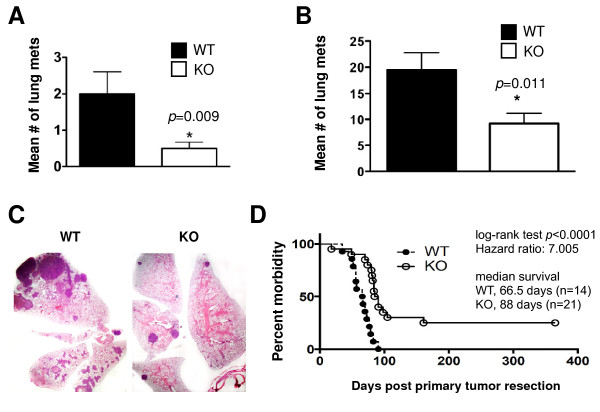
**HIF-1α expression in tumor epithelium is required for metastasis**. **(A) **Mean number of micrometastases ± SEM observed when lungs were harvested at the same time as the primary tumors (> 1,000 mm^3^) (*n *= 9 wild-type (WT) and 14 knockout (KO) recipients, *P < 0.05, unpaired Student's *t*-test). **(B) **Enhancement of lung metastases when WT and KO primary tumors were surgically resected at equivalent volumes (500 to 750 mm^3^), followed by 8 weeks of survival (*n *= 32 WT and 22 KO recipients, *P < 0.05, Student's *t*-test). **(C) **Representative images of lungs harvested from WT or KO mammary tumor epithelial cell recipients 8 weeks after tumor resection (original magnification, ×50). **(D) **The impact of HIF-1α expression in tumor cells on the survival of recipients following tumor resection. A subset of animals subjected to primary tumor resection was allowed to survive until moribund due to metastasis. The morbidity hazard ratio was 3.78 times higher when hosts bore WT tumors (*n *= 14 WT and 15 KO hosts, logrank test, *P *= 0.00039, 95% CI = 1.53 to 9.31).

Initially, metastasis was evaluated in WT or KO tumor recipients by harvesting lungs on the same day as the primary tumors. Because recipients bore a single tumor, in contrast to the transgenic model, in which all ten mammary glands become tumor-laden, very few metastases were present. The lungs from WT hosts exhibited a mean of two micrometastases, and the majority of lungs from KO hosts were devoid of metastases (Figure 5A).

One caveat with regard to interpreting these data is that the tumors developed by the KO cells were 60% smaller than the WT (Figure 5B). Therefore, to compare the effect on lung metastasis when WT and KO tumor volumes were equivalent, the primary tumor was resected during a survival surgical procedure when tumors were at least 1.5 cm in diameter with a corresponding volume range of 500 to 750 mm^3^. Animals subjected to survival surgery to resect the primary tumor were divided into two cohorts. In the first cohort, females were euthanized 8 weeks postsurgery. WT tumor cells developed large macrometastases visible to the naked eye, as well as several micrometastases visible by microscopic analysis, with a mean of 19 metastases per lung (Figures 5B and 5C). In contrast, lungs from KO tumor recipients contained 50% fewer metastases (Figures 5B and 5C), which were also smaller overall than the WT metastases. Furthermore, 50% of host mice transplanted with WT cells were deemed moribund and required euthanasia prior to the 8-week postsurgical period, whereas all hosts harboring KO cells lived the full 8 weeks. Therefore, although KO MTECs retain the capacity to complete the full metastatic program, metastasis is significantly suppressed.

In the second cohort subjected to the tumor resection paradigm, recipients were allowed to survive until moribund due to lung metastases (Figure 5D). Recipients bearing WT tumors succumbed to lung metastases sooner than KO recipients did (Figure 5D). The median survival increased from 66.5 days for WT recipients to 88 days for KO recipients (14 WT and 21 KO; logrank score, *P *< 0.001). Furthermore, about 25% of mice implanted with KO tumors (5 of 21) lived longer than 1 year and did not develop any lung metastases as evaluated by H & E staining of sections from their lungs.

### Deletion of *Hif1a *decreases tumorsphere formation

**Figure 6:**
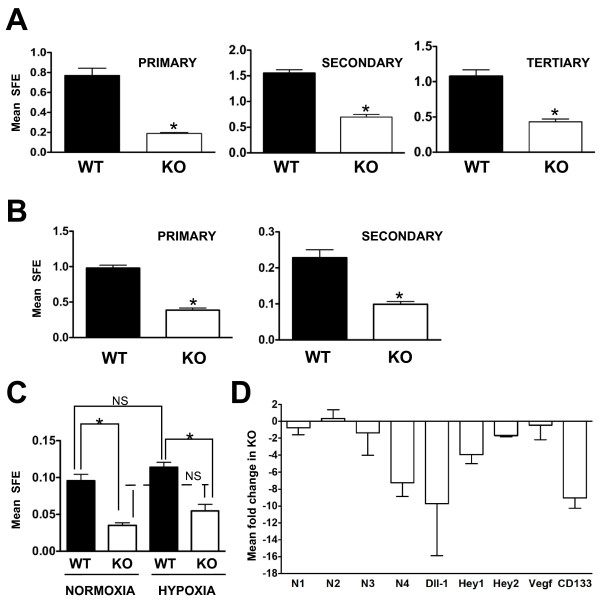
**HIF-1α promotes tumorsphere formation in cultured cells and in freshly isolated tumor cells independent of oxygen tension**. **(A) **Representative experiment in which the mean sphere formation efficiency (SFE) ± SEM was determined when wild-type (WT) and knockout (KO) cells were cultured in tumorsphere culture conditions. Tumorspheres ≥ 100 μm were scored positive, and the mean SFE (the percentage of cells capable of forming spheres per total number of single cells plated) per experiment (*n *≥ 12 wells/genotype, *P *< 0.05, unpaired Student's *t*-test) was determined at each of the primary, secondary and tertiary generations of spheres. A summary presenting the mean fold enrichment in WT SFE observed among biological replicates is included in Additional file 2 Figure S6. **(B) **A representative experiment in which the mean primary and secondary SFE ± SEM was determined when single cells were isolated from mammary tumors of *Hif1a DF*, MMTV-PyMT+ mice, followed by immediate adenoviral transduction (*n *≥ 12 wells/genotype, *P *< 0.05, unpaired Student's *t*-test). **(C) **Comparison of SFE when WT or KO cultured cells were subjected to normoxia or chronic hypoxia. Immediately following plating of single cells, dishes were cultured under normoxic or hypoxic conditions for the duration of the experiment; therefore, cells were exposed to chronic hypoxia. The graph presents mean SFE obtained from the secondary generation of spheres (n ≥ 12 wells/genotype). Differences among columns were assessed by one-way ANOVA, *P < 0.05. **(D) **The mean fold change in gene expression observed in KO tumorspheres was evaluated by qRT-PCR. The grand mean fold change ± SEM was determined on the basis of three independent experiments. cDNA was derived from three preparations of WT and KO secondary spheres grown in six-well format and pooled at the experiment end point to prepare total RNA (*n *= 3 pools of spheres/genotype). The mean expression of *Notch 4 *(N4), *Dll1, Hey1, Hey2 *and *Prom1 *(CD133) was reduced in KO tumorspheres by > 1.5-fold, whereas the mean expression of *Notch1, Notch2 *and *Notch3 *(N1 to N3) or *Vegf *did not vary by > 50% between genotypes. The decreased expression in KO tumorspheres is indicated as a negative fold change.

To begin to determine whether HIF-1α may play a role in regulating breast cancer stem cell activity, single WT or KO cells were placed into tumorsphere culture. All data shown in Figure 6 are representative of experiments with several biological replicates. A summary of the fold changes in SFE (the number of cells capable of forming spheres/total number of cells plated × 100) among biological replicate experiments is presented in Additional file 2 Figure S6. As shown in Figure 6A, the mean SFE decreased 4.1-fold in the primary, 2.2-fold in the secondary and 2.5-fold in the tertiary generations of spheres derived from KO cells, respectively. The mean SFE among biological replicate experiments varied from 0.06% to 1.55% for WT cells and from 0.015% to 0.85% for KO cells. Variability in the sphere assay among biological replicates by more than a log_10 _factor is not unusual [42]. More importantly, SFE within each experiment was always greater for WT cells than for KO cells.

As shown in Additional file 2 Figure S6, the mean fold enrichment in SFE in WT cells was 4.3-fold for the primary generation (range 2.85- to 7.23-fold), 4.1-fold for the secondary generation (range 1.85- to 6.7-fold) and 2.61-fold for the tertiary generation (range 2.1- to 2.79-fold). In each case, the mean fold change was determined on the basis of at least three biological replicates/genotype/generation. Because the WT and KO cells had previously been cultivated in monolayer in the presence of serum, the ability of HIF-1α to promote SFE was confirmed using tumor cells derived from freshly digested *Hif1a DF*, PyMT+ tumors originating in intact transgenic mice.

Following isolation from freshly digested tumors, single cells were immediately exposed in suspension to adeno-β-gal or adeno-Cre (moi of 80, for 2 to 3 hours), washed to remove viral particles and then plated at equal density into sphere culture conditions. Spheres derived from the primary generation were digested with trypsin, reexposed to adeno-β-gal or adeno-Cre in suspension (80 moi for 2 to 3 hours) and plated for second-generation spheres. Deletion efficiency was determined for each generation by qRT-PCR of genomic DNA isolated from a fraction of cells collected at 72 hours postplating, prior to the formation of large tumorspheres.

In contrast to the established KO MTECs, not all *floxed *cells exposed 2-3h to adeno-Cre were deleted for *Hif1a*. A mean deletion efficiency of 71% or 84% was observed following the first or second round of transduction with adeno-Cre, respectively. Despite incomplete *Hif1a *deletion primary and secondary SFE was reduced in *Hif1a*-deleted cells by 2.53-fold and 2.30-fold at the primary and secondary generations, respectively (Figure 6B). The mean fold change in SFE between WT and *Hif1a *deleted (KO) cells among three biological replicates of this assay was 5.74-fold for the primary generation (range of 2.53-8.2- fold) and 4.38-fold for the secondary generation (range of 2.30-6.54-fold) (Additional file 2 Figure S6). These results confirm that the enrichment in SFE observed for WT cells previously cultured in monolayer with FBS was not an artifact due to culture conditions, and suggested that HIF-1α may promote breast TIC potential *in vivo*.

Sphere formation was also assayed after culture of WT and KO cells at normoxia or chronic hypoxia. Figure 6C presents data from one experiment in which there was a 2.28-fold reduction in KO SFE at normoxia and a 2.1-fold reduction in KO SFE at hypoxia, as compared to WT cells. Among biological replicates the mean fold reduction in KO SFE at normoxia was 3.26-fold (range of 2.20-6.15-fold) and 6.85-fold at hypoxia (range of 2.1 to 9.17-fold) (Additional file 2 Figure S6). Although there was a trend towards hypoxia increasing WT SFE (mean fold increase in SFE of 1.38, and a range of 1.17- to 1.70-fold, Additional file 2 Figure S6), the increase was not statistically significant in any independent experiment by ANOVA. Changes in KO SFE between normoxia and hypoxia were also not significant (mean fold change of 0.94, and a range of 0.35-2.05-fold).

To determine if HIF-1α expression varied in spheres cultured acutely or chronically at hypoxia, HIF-1α expression was evaluated by western blotting of HS-WCE prepared from WT or KO spheres exposed to prolonged hypoxia (≥ 4 days), or from WT or KO spheres that were initially grown in normoxic culture, but were then briefly exposed acutely to hypoxia (6 h) prior to harvest (Additional file 2 Figure S7). In sphere culture conditions, HIF-1α was barely detectable in WT cells exposed to chronic hypoxia, whereas robust expression of HIF-1α was observed in WT cells exposed acutely to hypoxia. Therefore, in contrast to data obtained from monolayer cultured cells, EGF was not sufficient to stabilize HIF-1α expression when cells are grown constitutively at hypoxia in sphere culture.

One caveat of the tumorsphere assay is that spheres may be derived from aggregation of single cells, or even fusions of small spheres. The contribution of cell aggregation to sphere formation was not directly assessed in these experiments. However, the impact of aggregation on overall WT and KO SFE is presumed to be minimal since similar fold-changes in SFE between WT and KO cells were observed when cells were plated in sphere medium supplemented with 0.5% methylcellulose (data not shown), which acts as a physical barrier to impede cellular aggregation.

### HIF-1α-dependent regulation of CD133 and the Notch pathway

Since CD133, Notch receptors and Notch target genes may each be modulated by the HIF transcription factors [16,43,44], the expression of CD133, genes in the Notch pathway and classic HIF targets were evaluated in WT and KO tumorspheres by qRT-PCR. Figure 6D presents the mean fold reduction in gene expression observed in KO tumorspheres. Only genes that were consistently observed to be downregulated across three biological replicate experiments are shown. Expression of *Notch4 *was downregulated an average of 7.24-fold, whereas expression of the Notch receptors *Notch1, Notch2 and Notch3 *did not vary by more than twofold. The Notch ligand Delta-like-1 (*Dll1I*) was also downregulated in KO spheres an average of 9.73-fold, and the transcription factors *Hey1 *and Hey2 were downregulated 3.95-fold and 1.9-fold, respectively. The *HEY *family of genes has been proposed to function as surrogate markers of Notch activity in multiple human breast cancer cell lines [45,46].

Although the expression of the Jagged ligands has been reported to be hypoxia-inducible in breast cancer cell lines [45,46], we were unable to confirm a consistent effect of HIF-1α on the expression of either *Jagged1 *or *Jagged2 *by tumorspheres. For example, the expression of *Jagged1 *was upregulated 3.0-fold in two experiments and downregulated 3.0-fold in a third experiment, whereas the expression of *Jagged2 *was observed to be upregulated between 3- to 10-fold in two experiments and downregulated 3.0-fold in a third experiment (data not shown). In addition, the expression of *Dll4, Hes1, Hes2 *or *Vegf*, which is a known direct HIF target gene, did not vary between WT and KO spheres by more than 1.5-fold. The most striking difference in gene expression was observed for *Prom1 *(CD133), which was consistently downregulated > 9.0-fold in KO spheres (Figure 6D).

**Figure 7:**
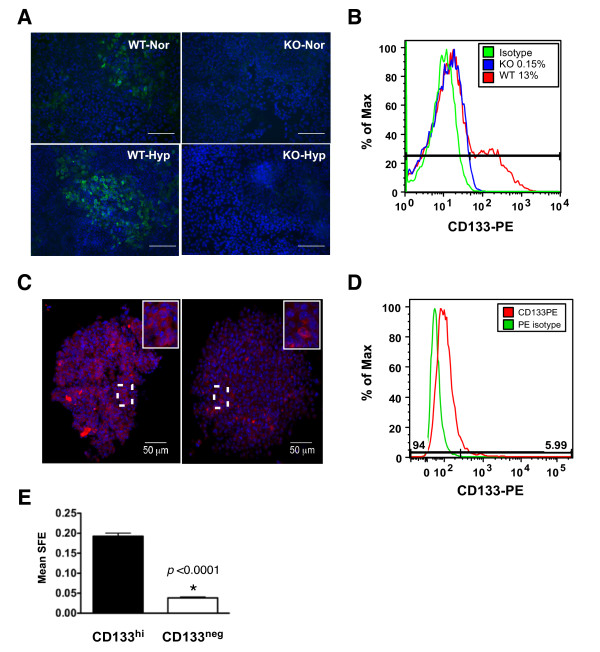
**CD133 expression in PyMT mammary tumor epithelial cells is regulated by HIF-1α**. **(A) **Subconfluent wild-type (WT) or knockout (KO) cultured cells were exposed to acute hypoxia (Hyp) (6 hours), then immunostained for CD133 (green) and counterstained with DAPI. Images were captured at ×200 original magnification. Scale bar = 50 μm. **(B) **Representative CD133-phycoerythrin (CD133-PE) staining profiles of WT (red histogram) and KO (blue histogram) cells cultured at normoxia (Nor) and subjected to fluorescence-activated cell sorting analysis. The isotype-only antibody control is also plotted (green histogram). The percentage of cells that were CD133-positive was determined on the basis of the live, singlet, hematopoietic lineage panel-negative (Lin^neg^) parent population using the FlowJo data analysis software package. **(C) **Secondary WT or KO tumorspheres cultured at normoxia were harvested at the study end point from ultralow adhesion wells, dried onto glass slides, stained with CD133-PE, counterstained with DAPI and imaged by confocal microscopy. The highlighted area (white boxes) shows a higher-magnification image, which demonstrates that CD133 is localized to the cell surface. **(D) **Histogram of CD133-PE (red) in the live, singlet, Lin^neg ^parent population of cells isolated from tumors that arose in the PyMT transgenic mouse as compared to the isotype antibody control (green). Approximately 6% of cells were defined in this experiment as CD133^hi^. **(E) **A representative experiment showing the mean sphere formation efficiency (SFE)/well ± SEM of CD133^hi ^versus CD133^neg ^subpopulations that were isolated by flow sorting and cultured at normoxia at a density of 10,000 cells/well in six-well format (*n *≥ 8 wells/genotype, unpaired Student's *t*-test). The SFE was determined as the percentage of cells capable of forming spheres per the total number of single cells plated.

On the basis of the qRT-PCR results, the expression of CD133 was then evaluated by immunostaining monolayer-cultured WT and KO MTECs grown at normoxia or hypoxia (Figure 7A). Compared to WT cells, CD133-positive cells were not readily detectable by immunostaining of KO cells following culture at normoxia or hypoxia (Figure 7A). These results were validated by flow cytometry following staining of WT and KO cells cultured at normoxia with CD133-PE antibodies. As shown in Figure 7B, the expression of CD133 was reduced in KO cells more than 9.0-fold. CD133 expression was also compared by immunostaining tumorspheres derived from WT and KO cells that were grown constitutively at normoxia (Figure 7C). As in cultured cells, the expression of CD133 was increased in WT spheres relative to KO spheres (Figure 7C), although in contrast to monolayer culture, CD133-positive cells were observed in KO spheres. Culture conditions influenced the expression of CD133 mRNA levels because expression increased by a mean of 5-fold in WT cells spheres relative to the same passage of WT cells cultured in monolayer in 2% FBS (data not shown).

To test whether CD133 expression influences SFE, spheres were derived from the CD133^hi ^or CD133^neg ^subpopulations of late-stage carcinomas arising in PyMT+ transgenic females. Live Lin^neg ^single cells were studied by flow cytometry after being stained with CD133-PE. Among replicate experiments, the tumor epithelial cells identified as highly positive for CD133-PE ranged from 5% to 12%. A representative histogram is presented in Figure 7D, showing that 5.99% of cells were isolated as the CD133^hi ^fraction. An example of the gating strategy applied to sorting experiments is outlined in Additional file 2 Figure S8. The mean increase in SFE in the CD133^hi ^subpopulation compared to the CD133^neg ^fraction among replicate experiments was > 4.0-fold (a representative experiment is shown in Figure 7E), suggesting that CD133 may be a useful cell surface marker to enrich for TICs in the PyMT model.

### HIF-1α promotes tumor-initiating cell frequency *in vivo*

On the basis of the dramatic differences in growth rates of WT and KO tumors and the reduction in SFE by HIF-1α-KO cells in tumorsphere assays, the ability of WT and KO cells to form tumors in recipients under limiting dilution transplantation conditions was evaluated. To permit estimation of TIC frequency due exclusively to loss of HIF-1α activity, TIC potential was compared using unsorted WT or KO MTECs to avoid the physical stress encountered during sorting that reduces cell viability and therefore affects the calculation of TIC frequency [20]. The ability of WT or KO MTECs to form a palpable tumor mass > 5 mm was evaluated using a broad range of cell dilutions (10 to 500 cells: 10, 25, 50, 100, 200 and 500) (Table 1).

**Table 1:** Estimation of tumor-initiating cell frequency of hypoxia-inducible factor-1α wild-type and knockout mammary tumor epithelial cells^a^

	MTEC genotype				
				
Estimated cell frequency	HIF-1α WT tumor-positive	HIF-1α-KO tumor-negative	HIF-1α WT	HIF-1α-KO	*P *values^b^
Number of cells injected, % (*n*)					
500	78% (7 of 9)	0% (0 of 8)			0.0023
200	90% (9 of 10)	10% (1 of 10)			0.0011
100	77% (10 of 13)	9% (1 of 11)			0.0003
50	69% (11 of 16)	6% (1 of 16)			0.0006
25	30% (6 of 20)	0% (0 of 20)			0.0202
10	35% (8 of 23)	0% (0 of 22)			0.0038
Estimated TIC frequency by ELDA (95% CI)			1 of 82 (1 of 57 to 1 of 117)	1 of 2,915 (1 of 925 to 1 of 9,193)	5.79e^-^21

^a^ELDA, Extreme Limiting Dilution Analysis; HIF-1α, hypoxia-inducible factor 1α; KO, knockout; MTEC, mammary tumor epithelial cell; TIC, tumor-infiltrating cell; WT, wild type. Frequency of tumors following limiting dilution transplantation of WT or KO MTECs is shown. The number of tumor-positive recipients per the total number of recipients at day 36 posttransplant and the *P*-values between genotypes for each cell density tested are listed. The estimated frequency of TICs was determined using the publicly available ELDA software. ^b^*P *values were calculated using Fisher's exact test.

**Figure 8:**
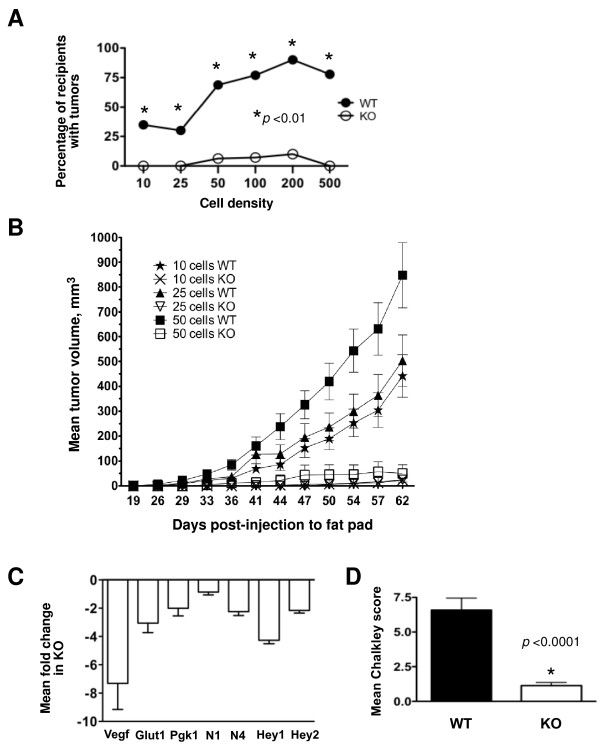
**HIF-1α regulates tumor initiation potential *in vivo***. **(A) **Evaluation of the percentage of recipients (number of recipients positive for a palpable mass per number of recipients successfully transplanted × 100) that developed a tumor mass by day 36 postinjection of unsorted wild-type (WT) and knockout (KO) tumor cells. The asterisks indicate a significant difference between genotypes for a given cell density by Fisher's exact test. **(B) **Comparison of WT and KO mean tumor volumes ± SEM over time when 10, 25 or 50 cells were injected into the mammary fat pads. **(C) **The mean fold change ± SEM in gene expression profiles of early-stage tumor masses was evaluated by qRT-PCR. All genes displayed were downregulated in KO tumors as compared to WT tumors (*n *= 4 tumors/genotype/experiment).

At each cell density evaluated, there was a statistically significant enrichment in TIC activity in WT cells, which was indicated by the percentage of FVB/Nj recipients positive for a palpable tumor (Figure 8A and Table 1). ELDA analysis [35] revealed a 35-fold reduction in TIC frequency from KO to WT cells at day 36 posttransplant (Table 1). Data were also statistically significant on the basis of the Fisher's exact test (Table 1). The difference in TIC potential remained statistically significant at day 62 posttransplant (Additional file 2 Figure S9, and Additional file 1 Table S3), with a 7.5-fold enrichment in TIC frequency in WT cells. One reason for the apparent decrease in the fold enrichment of TIC frequency at day 62 may be that the data from the 500- and 200-cell recipients were excluded from the ELDA analysis. All recipients in these two cohorts were euthanized at day 36 (Additional file 2 Figure S10) to compare the histological and gene expression profiles of early-stage lesions. Notably, WT recipients in the 50- and 100-cell input cohorts required surgical intervention or euthanasia based on tumor volume by day 62 (Additional file 2 Figures S11 and S12), whereas no intervention was required for KO recipients until day 96 posttransplant.

The mean volume of KO tumors derived under limiting dilution conditions was reduced, and this difference was maintained over several months. None of the KO tumors derived from transplantation of 50 or fewer cells exceeded a volume of 100 mm^3 ^by day 62 (Figure 8B). By day 112 posttransplant, 96% of recipients that received 10 WT cells had developed measurable tumors, whereas 55% of recipients that received 10 KO cells had not (Additional file 1 Table S4). The mean tumor volume among the tumor-positive 10-cell KO recipients was < 250 mm^3 ^(Additional file 2 Figure S12). Supplementary information summarizing the mean tumor volume per cohort and the percentage of recipients with measurable tumors at day 112 or 244 is presented in Additional file 1 Tables S4 to S7, and in Additional file 2 Figures S11 and S12. The difference in TIC potential in the 10-cell cohort remained significant until day 244 (Additional file 1 Table S7), when all animals were euthanized.

HIF-1α-dependent changes in gene expression were evaluated in early-stage WT and KO masses (75 to 200 mm^3^; five tumors per genotype). Similar to results obtained from spheres, *Notch4, Hey1 *and *Hey2 *mRNA levels were decreased in KO tumors 2.25-, 4.27- and 2.3-fold, respectively (Figure 8C), whereas *Notch1-Notch3 *mRNA levels decreased by less than 50%. All other genes in the Notch pathway that were previously found to be differentially expressed in spheres were not differentially expressed among WT and KO tumors, except for *Vegf*, which was downregulated by a mean of 7.3-fold. As observed in end-stage tumors, WT and KO *Glut-1 *and *Pgk-1 *mRNA levels were reduced in tumors < 200 mm^3^.

No gross changes in WT or KO tumor histopathology were observed in recipients transplanted with 500 tumor cells. None of the KO masses within this cohort were detectable by manual palpation, but microscopic analysis of H & E-stained sections revealed lesions < 5 mm in five of eight recipients. Since *Vegf *mRNA was differentially expressed, microvessel density was evaluated by Chalkley scoring of sections immunostained with anti-CD34 antibodies. Decreased *Vegf *mRNA levels corresponded with a concomitant decrease in mean Chalkley score in KO tumors (Figure 8D). In summary, deletion of *Hif1a *reduced TIC potential as well as neoangiogenesis.

## Discussion

Although the role of hypoxia and the HIF-1α transcriptional response in promoting tumor progression and metastasis is well-established, the direct contribution of the HIF family to the regulation of TICs in breast cancer is unknown. HIF-1α rather than HIF-2α is the predominant regulator of the hypoxic response in breast cancer [47]; therefore, we sought to determine the effect of *Hif1a *deletion in the MMTV-PyMT model of breast cancer. In this study, by using an *ex vivo *genetic deletion approach, we generated constitutive HIF-1α-KO and control (WT) MTECs isolated from a pool of MMTV-PyMT tumors. One distinct advantage of this approach is that the MTECs are transplantable to immunocompetent hosts, preserving any host-derived effects on TIC potential following limiting dilution transplantation, in contrast to the xenograft of human cells into immunocompromised mice. Herein we show for the first time that HIF-1α positively regulates TIC activity in breast cancer as suggested by sphere formation assays *in vitro *and validated through limiting dilution transplantation of WT and KO cells.

Additional advantages of the exogenous transduction and transplantation approach versus crosses to MMTV-Cre-transgenic mice include avoiding the mosaic nature of MMTV-Cre expression, because not all epithelial cells harboring *floxed *alleles of *Hif1a *undergo recombination [48], and avoiding the use of a mixed genetic strain background by utilizing mouse models backcrossed to a single strain (FVB/Nj). Because *Hif1a *deletion impairs cell proliferation [39], an effect we also observed in cultured KO MTECs (Figure 1B), incomplete recombination could permit nontargeted cells to outgrow the recombined (KO) cells. Additionally, it had not previously been assayed whether the lung metastases originating in the MMTV-Cre-derived conditional KO females were derived from the recombined tumor cells or were generated from cells that had escaped recombination [27]. In addition, the prior use of a mixed strain background may have introduced genetic modifier effects that could influence tumor incidence and lung metastasis. In particular, both tumor burden and lung metastasis in the MMTV-PyMT model are enhanced on the FVB/N background as compared to C57BL/6 [49].

In agreement with the findings of previous studies, our study results confirm that HIF-1α promotes the growth of MTECs cultured at hypoxia and enhances lung metastasis *in vivo *[27]. However, there were some differences observed between the two model systems. For example, we found that deletion of *Hif1a *repressed the growth of cells at normoxia as well as at hypoxia (by 48 hours of culture). In addition, the magnitude of the decrease in KO cell invasion was similar, regardless of whether cells were cultured at normoxia or hypoxia, whereas Liao *et al*. reported a difference only at hypoxia [27]. No statistically significant increase in invasion potential between WT cells cultured at normoxia and hypoxia was observed, as reported previously [27]. In contrast to observations from either PyMT model system in which *Hif1a *was deleted, short hairpin RNA-mediated knockdown of *HIF1A *in MDA-MB-231 breast cancer cells does not significantly change cell number at either normoxia or hypoxia (1% O_2_) [50]. It is possible that the effects of loss of HIF-1α activity on PyMT cell growth in monolayer culture would be attenuated if cells were cultured at higher serum levels since MDA-MB-231 cells were cultured in medium containing 10% FBS whereas PyMT cell lines were cultured in medium containing 2% or 5% FBS.

We further demonstrate that primary tumor growth and survival from distant metastases are dependent upon epithelial cell intrinsic HIF-1α expression. In stark contrast to results obtained previously [27], we found that HIF-1α plays a significant role in the control of primary PyMT-induced mammary tumor growth under either standard (50,000-cell input) or limiting dilution cell transplantation conditions (50- to 500-cell input). The cause of the control of net mammary tumor growth by HIF-1α is unclear, although our data suggest that HIF-1α-dependent control of TIC activity may be a primary mechanism driving tumorigenesis. The significant changes in *Vegf *mRNA expression and microvessel density observed in early-stage KO tumors may also contribute to the phenotype by furthering restricting tumor growth once tumors are initiated. In agreement with our observations, a HIF-1α-dependent effect on tumor growth was demonstrated recently in both MDA-MB-231 and MDA-MB-435 breast cancer xenografts [50,51].

Characterization of the expression of luminal (K8/K18) and myoepithelial (K14/K5) lineage markers in WT and KO PyMT cells and tumors revealed that fewer KO cells than WT cells coexpressed K8/K14, primarily due to loss of K14-positive cells. These results suggest that loss of HIF-1α activity corresponds with a reduction in bipotent, stemlike cells in PyMT tumors. The most dramatic phenotype was the absence of K5-positive cells in KO cultures and the loss of both K14- and K5-positive cells in KO tumors. These data are of interest, given the stratification of triple-negative breast cancers (TNBCs) into non-basal-like or basal-like subtypes, based on the expression of both K5 and epidermal growth factor receptors (EGFRs) in tumors within the basal-like subtype [52].

TNBC patients with a basal-like classification have shorter disease-free or overall survival [53,54] and tumors from TNBC patients with metastatic disease exhibit higher levels of K5 and EFGR [55]. A potential influence of HIF-1α on the ability of the K5 and EGFR biomarkers to predict disease-free survival in basal-like TNBC is intriguing in light of our observations that, in cultured cells, EGF stabilizes HIF-1α expression at normoxia and further potentiates hypoxia-inducible expression of HIF-1α. Although the PyMT model is classified as a luminal-like cancer [26], late-stage tumors such as the ones utilized to generate HIF-1α WT and KO cells are ER- [25]. Subpopulations of both WT cells and WT tumors were positive for K5.

It has also been observed in luminal breast cancer cell lines (T47D and MCF7), which contain subpopulations of ER-/PR-/K5+ cells, that the K5+ cells are enriched for TIC activity and are resistant to conventional chemotherapies compared to ER+/PR+/K5- cells [56]. Of additional relevance to our results regarding the decreased expression of K5, K14 and markers of EMT, including *Slug*, by KO tumors is that hypoxia-dependent elevation in K5 mRNA levels occurs in a SLUG-dependent manner in MCF7 cells [40]. Furthermore, a statistically significant correlation was found to exist between tumors with high *SLUG *expression and *PROM1 *(CD133) expression. These tumors also expressed high levels of carbonic anhydrase IX (*CAR9)*, which is a known HIF-1 target gene [40].

Recent whole-genome expression profiling of breast cancers has revealed that the hypoxic response (predominantly through HIF-1α), the EGFR and signal transducer and activator of transcription 3 (STAT3) pathways are positively correlated together in TNBCs as compared to luminal cancers [57]. In support of a functional association between the HIF-1α and EGFR pathways in TNBCs, MDA-MB-231 cells treated with gefitinib were found to exhibit downregulation of HIF-1α transcriptional activity that corresponded with decreases in cell viability and migration, whereas resistance to cetuximab or lapatinib therapy was hypothesized to be due to the inability of either drug to downregulate HIF-1α activity [56]. Taken together, these observations suggest that targeting the HIF pathway may be beneficial to TNBC patients, particularly those patients diagnosed with basal-like TNBC.

Based upon the HIF-1α-dependent control of CD133 expression in MTECs and tumorspheres, as well as the enrichment of sphere formation in the CD133^hi ^versus CD133^neg ^populations, we have identified CD133 as a cell surface marker that may enrich for TICs in the PyMT model. Antibodies to epitope 2 of CD133 (CD133/2) have been utilized extensively to enrich for TICs in other solid tumors, particularly in human colon cancer and gliomas. In contrast, in the normal mammary gland, CD133 is expressed by differentiated ER+ luminal cells, and CD133+ cells exhibit lower regenerative capacity than CD133- cells [58]. Notably, deletion of *Prom1 *in a knockout mouse model did not impair the regenerative capacity of the normal mammary gland, but did reduce ductal branching during morphogenesis by increasing the ratio of luminal to basal cells [59]. Yet, in the NKI 295 data set, *PROM1 *expression levels were found to be lower in ERα+ tumors than in ERα- tumors [59]. The association of HIF-1α and *PROM1 *expression in ER- breast cancers is not surprising, given that hypoxia is a potent stimulator of ERα degradation [60,61].

In contrast to observations in the normal mammary gland, Meyer and colleagues have recently shown that, in the context of ER- breast cancers, CD133 enriches for TICs when used in conjunction with CD49f (integrin α6) and CD44 [62]. Specifically, the CD49f+/CD44+/CD133^hi ^population identified tumor cells with enriched sphere-forming and xenografting potential. Likewise, CD133 has also been shown to enrich for TICs in the *Brca1 *conditional mouse [21]. That a marker of a differentiated normal mammary epithelial cell could enrich for cells with TIC activity in the context of breast cancer is also supported by recent evidence from multiple laboratories that, in *BRCA1 *basal-like tumors, the TIC population arises from the luminal lineage [63-65]. Interestingly, basal-like *BRCA1 *tumors have previously been shown to overexpress HIF-1α and higher HIF-1α levels have been found to be correlated with decreased disease-free survival [66,67]. Moreover, Proia *et al*. found that SLUG promoted a basal-like phenotype before and after transformation in *BRCA1 *tumors [65], which is consistent with our observation that expression of *Slug *decreased more than threefold in HIF-1α-KO PyMT tumors that did not express the basal markers K5 and K14.

Furthermore, accumulating evidence suggests that differentiated cells (lineage-restricted progeny) may reacquire stem cell-like potential and tumor-initiating capacity rather than follow a strict linear hierarchy as originally proposed for the normal mammary gland. The plasticity involved in breast stem cell biology is emerging. Two independent groups have observed the spontaneous conversion of non-stem cells into stem cells [68,69]. Likewise, the results of recent lineage-tracing experiments by researchers in the Blanpain laboratory have challenged the requirement for a bipotent stem cell in the postnatal normal mammary gland [70]. Under the limiting cell conditions routinely used to document the regenerative capacity of a given cell population, these authors found that the disruption of the normal luminal-to-myoepithelial cell ratio is sufficient to stimulate a unipotent myoepithelial progenitor cell to reacquire a bipotent progenitor activity that is normally restricted to the embryonic gland [70]. Yet, how, or if, this model derived from lineage-tracing experiments can be applied to TICs during breast tumorigenesis remains unknown.

One mechanism of HIF-1α-dependent control of TIC may be through regulation of the Notch pathway. Interactions of HIF-1α with the Notch intracellular domain enhances the regulation of Notch transcriptional targets, such as the *HEY *genes, and promotes EMT in breast cancer [16,71,72]. In addition, in breast cancer, NOTCH1 and NOTCH4 have been positively correlated with stemness, and blocking antibodies to NOTCH4 reduces mammosphere formation [72]. In KO tumorspheres and early-stage KO tumors, decreased expression of several members of the Notch pathway, particularly *Notch4 *and *Hey1*, was observed. Changes in *Notch4 *are of particular interest because previous studies have shown that blocking NOTCH4 receptor activity inhibits tumor formation of xenografted breast cancer cells, whereas blocking NOTCH1 has less of an effect [72]. Although a positive correlation between hypoxia and *NOTCH3 *expression was previously described in breast cancer [73], no HIF-1α-dependent changes in *Notch3 *were observed in our studies.

HIF-1α-dependent effects on sphere formation efficiency *in vitro *and TIC activity *in vivo *were observed using parental tumor cells without first enriching for a putative CSC subpopulation based on cell surface markers. One rationale for this approach is the lack of comprehensive information on the markers that define TICs in the PyMT model. In a similar PyMT tumor cell transplant paradigm to ours, the population of CD24 (heat stable antigen)^hi^/CD29(β_1_-integrin)+/CD61(β_3_-integrin)+ cells was found to significantly increase during tumor progression, specifically at the transition from hyperplasia to carcinoma. Greater than 90% of cells were characterized as CD24^hi^/CD29+/CD61+ in late-stage carcinomas, and this population also had enhanced invasive potential *in vitro *[19]. More recently, researchers in the Visvader laboratory have shown that, in cells derived from PyMT adenomas (early-stage lesions), CD14 and c-kit, along with CD49f/CD24, enriches for cells with colony-forming potential [74].

In addition, the physical stress of flow sorting decreases cell viability, which therefore directly influences the estimated TIC frequency as determined through limiting dilution transplantation. As observed for the MMTV-Neu model, the TIC frequency of unsorted cells was 1 in 61, decreasing to 1 in 177 for cells enriched by sorting [20]. Likewise, for cells isolated from PyMT adenomas, the TIC frequency was estimated to be 1 in 556 for unsorted cells, whereas the TIC frequency in Lin^neg ^cells following sorting for CD24 was 1 in 648 [74]. Using unsorted cells isolated from late-stage carcinomas of the PyMT model, we observed a TIC frequency of 1 in 82. It is possible that the TIC frequency in the PyMT model may vary based upon both the stage of progression and subtle differences in technical procedures among laboratories.

The specific cell surface markers that enrich for breast tumorsphere or TIC activity are also likely to vary in each mouse model. In the Balb/C p53^-/- ^model, mammary tumor cells double-positive for CD24 and CD29 were found to exhibit TIC activity [22]. In contrast, in the MMTV-Neu model Sca-1+ cells were found to correlate with sphere formation [75], and CD61 was also described as a marker that enriched for TICs [20]. How HIF-1α directly affects various tumor cell subpopulations defined through flow cytometric profiling for the known murine mammary stem cell markers, or whether HIF-1α is preferentially expressed in a given subpopulation requires further extensive investigation.

## Conclusions

Tumor hypoxia profoundly affects all aspects of tumorigenesis, including tumor growth, angiogenesis, metastasis and response to chemotherapy and radiation. Although HIF's effects are pleiotropic, evidence for the role of HIF-dependent TICs in controlling these phenotypes and the solid tumor stem cell niche is increasing. Notably, a recent report indicated that oxygen tension can profoundly influence TICs because repetitive cycles of hypoxia and reoxygenation promoted breast cancer cell lines to permanently acquire stemlike properties [76]. Our studies demonstrate that the hypoxic response, specifically through HIF-1α, is important for controlling breast cancer stem cell behavior through the regulation of CD133 and the Notch pathway. These data, together with previous observations that the HIFs directly mediate glioma, lymphoma and AML TIC activity [15,16], suggest that attenuation of HIF activity may effectively eradicate TICs in a variety of cancers, leading to improved therapeutic response and overall survival.

We acknowledge the possibility that HIF-1α may regulate activity of TICs and differentiated cell lineages simultaneously within a tumor. For example, HIF-1α may regulate breast TIC activity in a stemlike cell population expressing CD133, but HIF-1α activity may also be required by differentiated cells that may act to support TICs through paracrine signaling, perhaps via a HIF-dependent secreted growth factor such as VEGF. Whether the EGFR pathway is required for HIF-1α-dependent regulation of TIC activity, particularly in basal-like TNBC, also requires further investigation. Given the pleiotropic role of HIF-1α in tumorigenesis and metastasis, as well as the increasing evidence that stem cells may evolve *de novo *from non-stem cells, targeting both the TIC and non-TIC ("bulk") populations in breast cancer is likely necessary to successfully treat primary breast cancer and to prevent metastasis.

## Abbreviations

AML: acute myeloid leukemia; CSC: cancer stem cell; DAPI: 4',6-diamidino-2-phenylindole; DMEM: Dulbecco's modified Eagle's medium; ELDA: Extreme Limiting Dilution Analysis; ES: embryonic stem; FBS: fetal bovine serum; H & E: hematoxylin and eosin; HSC: hematopoietic stem cell; KO: knockout; Lin: hematopoietic lineage panel; MTEC: mammary tumor epithelial cell; NBF: neutral buffered formalin, 10%; PBS: phosphate-buffered saline; PCR: polymerase chain reaction; PFA: paraformaldehyde, PyMT: polyoma virus middle T; qRT-PCR: quantitative real time polymerase chain reaction; TIC: tumor-initiating cell; TNBC: triple-negative breast cancer; WCE: whole-cell extract; WT: wild type.

## Competing interests

The authors declare that they have no competing interests.

## Authors' contributions

LPS participated in the study design, created the MTECs, carried out the animal studies, performed the tumorsphere assays using MTECs and did the Western blot analysis and qRT-PCR. She also performed the statistical analyses, assisted with creating the figures and wrote the manuscript. DLP carried out the animal studies, flow cytometry sorting, immunostaining, Western blot analysis, qRT-PCR assays and statistical analyses and assisted with creating the figures and editing the manuscript. LPS and DLP contributed equally to the study. DM performed the tumorsphere assays using freshly digested tumors, performed qRT-PCR and analyzed the data. KDS optimized immunostaining conditions and assisted with drafting figures. JFI performed and analyzed the cell invasion assays, genotyped the animals and cultured cell lines derived from the animals. RCC and LCJ performed tumor resection surgery, assisted with limiting dilution transplantation surgeries, prepared lung sections, counted metastases and analyzed metastatic data. TNS conceived the study, created the study design, assisted with animal surgery and data analysis, created the figures and drafted the manuscript. All authors read and approved the final manuscript.

## Supplementary Material

Additional file 1 **Table S1 Primers and Roche Universal Probe Library (UPL) 6-carboxyfluorescein-labeled probes utilized in quantitative RT-PCR assays**. **Table S2 Primary antibody source and dilution factors utilized in Western blotting, immunohistochemistry (IHC), immunofluorescence (IF) and fluorescence-activated cell sorting (FACS)**. Anti-α-SMA, anti-α-smooth muscle actin; anti-mouse CD133-PE, anti-mouse CD133-phycoerythrin; anti-mouse ERα, anti-mouse estrogen receptor α; anti-mouse CD24-FITC, anti-mouse CD24-fluorescein isothiocyanate; HRP, horseradish peroxidase; SA-APC, streptavidin-allophycocyanin. **Table S3 Frequency of tumors in recipient mice at day 62 after limiting dilution transplantation**. ELDA, Extreme Limiting Dilution Analysis; HIF-1α, hypoxia-inducible factor 1α; KO, knockout; MTEC, mammary tumor epithelial cell; TIC, tumor-initiating cell; WT, wild type. **Table S4 Frequency of tumors in recipient mice at day 112 after limiting dilution transplantation**. ELDA, Extreme Limiting Dilution Analysis; HIF-1α, hypoxia-inducible factor 1α; KO, knockout; MTEC, mammary tumor epithelial cell; TIC, tumor-initiating cell; WT, wild type. **Table S5 Summary of the percentage and total number of recipients bearing small tumors at day 112 posttransplant**. KO, knockout; WT, wild type. **Table S6 Frequency of tumors in recipient mice at day 244 after limiting dilution transplantation**. ELDA, Extreme Limiting Dilution Analysis; HIF-1α, hypoxia-inducible factor 1α; KO, knockout; MTEC, mammary tumor epithelial cell; TIC, tumor-initiating cell; WT, wild type. **Table S7 Summary of the percentage and total number of recipients bearing small tumors at day 244 posttransplant**. KO, knockout; WT, wild type. Click here for file

Additional file 2 **Figure S1 HIF-1α expression increase during tumor progression in the MMTV-PyMT mouse model and confirmation of HIF-1α deletion**. HS-WCE protein extracts were prepared from two individual transgenic MMTV-PyMT female mice (FVB/Nj strain) between 6 and 9 weeks of age. HS-WCE, high-salt whole-cell extract; MMTV-PyMT, mouse mammary tumor virus polyoma virus middle T. By palpation, each mouse had at least one mammary gland with hyperplastic (HP) (palpable as grainy), early-carcinoma (EC) (< 250 mm^3^) or late-carcinoma (LC) (> 500 mm^3^) lesions, which was expected because tumor progression among glands is asynchronous in this model. Two independent glands (one from each mouse) per stage were utilized to prepare HS-WCEs for Western blotting. As shown, HIF-1α expression generally increased during progression and was most abundant in late-stage carcinomas. Included as controls were HS-WCEs prepared from wild-type (WT) and knockout (KO) mammary tumor epithelial cells (MTECs) grown to 80% confluence, followed by hypoxia exposure for 6 hours at 0.5% O_2_. A cross-reactive material (CRM) band at about 76 kDa was detectable when lot E2 of NB 100-479 of the anti-mouse HIF-1α primary antibody (Novus Biologicals, Littleton, CO, USA) was used. KO cells were transduced twice with adenovirus-Cre in monolayer culture at 80 to 100 plaque-forming units/cell to achieve > 99% deletion efficiency as determined by qRT-PCR and Western blot analysis. **Figure S2 HIF-1α expression increases in response to EGF treatment at normoxia, and EGF prolongs HIF-1α stabilization at hypoxia**. Wild-type (WT) mammary tumor epithelial cells (MTECs) were either cultured in complete growth medium (containing 5% fetal bovine serum, FBS + 5 μg/ml insulin + 10 ng/ml epidermal growth factor, EGF) or in medium supplemented only with 2% FBS for at least five passages prior to replating to test the effect of EGF treatment on HIF-1α expression. Cells grown to 80% confluence were incubated at hypoxia for the number of hours indicated (3, 6 or 24). All cells were exposed to hypoxia beginning 24 hours prior to harvest (the *t *= 0 time point) such that cells at the *t *= 0 time point (normoxic control) were harvested at the same time as the *t *= 24 hours hypoxia time point. Cells were exposed to hypoxia for the indicated number of hours prior to removal from culture and the immediate preparation of high-salt enriched whole-cell extracts. HIF-1α was detected using lot E2 of NB 100-479 (Novus Biologicals, Littleton, CO, USA). **Figure S3 Histology of HIF-1α WT and KO end-stage PyMT tumors**. H & E-stained sections of end-stage tumors (> 750 mm^3^) derived from wild-type (WT) or knockout (KO) cells are shown. HIF-1α, hypoxia-inducible factor 1α; PyMT, polyoma virus middle T. The WT tumors typically contained more extensive areas of necrosis than the KO tumors. One representative tumor per genotype was imaged using 5 ×, 20 × and 63 × lens objectives of a Leica DM6000 upright microscope (Leica Microsystems, Buffalo Grove, IL, USA) with a SPOT camera (SPOT Imaging Solutions, Sterling Heights, MI, USA) for final original magnifications of ×50, ×200 or ×630, respectively. Scale bar = 50 μm. Digitally scanned whole slides of H & E-stained WT and KO tumor sections were captured using the Aperio ScanScope system (Aperio, Vista, CA, USA). Whole-slide images have been posted to a public database, which may be accessed using instructions provided in the Additional methods. **Figure S4**. **Ki67 and activated caspase 3 immunostaining in end-stage PyMT tumors**. Paraffin-embedded, formalin-fixed tissue sections from wild-type (WT) and knockout (KO) tumors (> 500 to 750 mm^3^) were immunostained with either Ki67 or activated caspase 3 antibodies, and immunoreactive complexes were detected using the VECTASTAIN Elite ABC Kit (Vector Laboratories, Burlingame, CA, USA) and developed with ImmPACT DAB substrate (diaminobenzidine; Vector Laboratories), followed by counterstaining with Harris hematoxylin. PyMT, polyoma virus middle T. Scale bar = 100 μm. **Figure S5 Expression of p63 and ERα in WT and KO end-stage PyMT tumors**. Representative results of p63 and estrogen receptor α (ERα) immunostaining in wild-type (WT) and knockout (KO) tumors (*n *= 4/genotype), along with expression observed in normal virgin mammary glands, which served as the positive control (*n *= 2 FVB strain mice about 8 weeks of age). HIF-1α, hypoxia-inducible factor 1α. Original magnification, ×200; scale bar = 50 μm. Black arrows point to examples of positive cells. No obvious changes in p63 expression or localization were detected between WT and KO tumors, whereas p63 was located basally within the virgin mammary ductal tree, as expected. No immunoreactivity for ERα was detected in end-stage WT or KO tumors (which were derived from late-stage carcinomas of the MMTV-PyMT transgenic mouse), whereas almost all ductal epithelial cells in the virgin normal mammary gland expressed ERα as previously reported [77]. **Figure S6 Overview of the mean fold enrichment in SFE in WT cells compared to KO cells**. **(A) **to **(C) **Bar graphs represent the mean fold enrichment of sphere formation efficiency (SFE) ± SEM for each generation (three biological replicates per genotype per generation in (A) and (B)) or for specific genotype-oxygen tension comparisons (C). Adeno-Bgal, adenovirus β-galactosidase; DF, double *floxed*; HIF, hypoxia-inducible factor 1α; KO, knockout; PyMT+, polyoma virus middle T-positive; WT, wild type. **(C) **The mean fold change in SFE observed for specific comparisons between genotype and oxygen tension is presented (nor, normoxia; hyp, hypoxia) (wild-type normoxia (WT nor) to knockout (KO) nor, *n *= 4; WT hypoxia (WT hyp) to KO hyp, *n *= 3; WT nor to WT hyp, *n *= 5; KO nor to KO hyp, *n *= 3). The SFE is a percentage and was determined as follows: [(number of cells capable of forming spheres)/(total number of single cells plated) × 100]. **Figure S7 HIF-1α expression in tumorspheres cultured acutely or chronically at hypoxia**. Wild type (WT) or knockout (KO) tumorspheres were originally derived from single cells plated into ultralow adhesion dishes and cultured at normoxia. HIF-1α, hypoxia-inducible factor 1α. A subset of WT and KO tumorspheres (80 to 100 μm in size) were then transferred from normoxic to hypoxic culture (0.5% O_2_) for the duration of incubation, which was either acute (6-hour exposure) or chronic (> 4 days' exposure). Spheres were then isolated, high-salt whole-cell extract (HS-WCE) was prepared and HIF-1α expression levels were evaluated by Western blot analysis (input of 1 μg HS-WCE/lane) using lot M1 of Novus 100-479 (Novus Biologicals, Littleton, CO, USA), which produced more cross-reactive material (CRM) bands than previously observed with lot E2. **Figure S8 Gating strategy for sorting CD133^hi ^versus CD133^neg ^populations from mammary tumors originating in PyMT+ transgenic females**. After being stained, cells resuspended in flow buffer were subjected to flow sorting on a FACSAria cell sorter (BD Biosciences, San Diego, CA, USA) at the University of Tennessee Health Science Center Flow Cytometry and Sorting Core. Forward scatter (FSC) and side scatter (SSC) gates were set to exclude particulate debris, followed by gating for singlets based on FSC area (FSC-A) and FSC height (FSC-H). Singlet events were determined to be viable by gating against cells that stained positive with SYTOX Blue (Molecular Probes/BD Biosciences, Eugene, OR, USA) or 7-AAD (BD Pharmingen, San Diego, CA, USA). Lin^neg ^live singlets were selectively gated based on the absence of expression of the mouse lineage marker panel (BD Biosciences) that was supplemented with CD31 (BD Biosciences) to exclude endothelial cells. The gating to enrich for CD133-PE^hi ^cells was manually set based on the isotype matched control antibody. PyMT+, polyoma virus middle T-positive; APC, allophycocyanin; IgG, immunoglobulin G; Lin, hematopoietic lineage panel; PE, phycoerythrin. **Figure S9 Comparison of WT and KO tumor-initiating potential at day 62 posttransplant**. The percentage of FVB recipients (tumor-positive recipients/total number of recipients successfully transplanted) that developed a palpable mass ≥ 5 mm in diameter was compared on day 62 after injection of wild type (WT) or knockout (KO) cells. The asterisks indicate a statistically significant difference between genotypes per cell density determined by Fisher's exact test. **Figure S10 Mean WT and KO tumor volume in the 200- and 500-cell input cohorts up to day 36 posttransplant**. Comparison of mean tumor volume ± SEM when 500 or 200 wild type (WT) or knockout (KO) cells were injected into a single, cleared inguinal mammary fat pad per recipient. All recipients in the 500- and 200-cell input cohorts were euthanized on day 36 posttransplant to obtain tissue sections and RNA during the early stages of tumor outgrowth. The number of recipients with palpable masses per genotype per density is reviewed in Table 1. **Figure S11 Mean tumor volume in the 100-cell WT and KO cohorts up to day 112 posttransplant**. Comparison of wild-type (WT) and knockout (KO) mean tumor volume ± SEM over time when 100 cells were injected into a single, cleared inguinal mammary fat pad. The decrease in mean tumor volume observed at day 112 for the 100-cell KO group is due to the exclusion of a subset of individual recipients in this cohort that developed tumors > 500 mm^3^. These recipients were subjected to surgical resection of the primary tumor between days 96 and 112 posttransplant. **Figure S12 Mean WT and KO tumor volume in the 10-, 25- and 50-cell input cohorts up to day 112 posttransplant**. Comparison of wild type (WT) and knockout (KO) mean tumor volume ± SEM over time when 10, 25 or 50 WT or KO cells were injected into a single, cleared inguinal mammary fat pad. The decrease in mean tumor volume observed at day 112 for the 100-cell KO group is due to the exclusion of a subset of individual recipients in this cohort that developed tumors > 500 mm^3^. These recipients were subjected to surgical resection of the primary tumor between days 96 and 112 posttransplant. Click here for file
