# Supplementary material for: Hypoxia-inducible factor 1α promotes primary tumor growth and tumor-initiating cell activity in breast cancer
Source: Breast Cancer Res. 2012 Jan 7;14(1):R6. doi: 10.1186/bcr3087 (PMC3496121; doi:10.1186/bcr3087)
Supplement: Additional file 1 — Table S1 Primers and Roche Universal Probe Library (UPL) 6-carboxyfluorescein-labeled probes utilized in quantitative RT-PCR assays. Table S2 Primary antibody source and dilution factors utilized in Western blotting, immunohistochemistry (IHC), immunofluorescence (IF) and fluorescence-activated cell sorting (FACS). Anti-α-SMA, anti-α-smooth muscle actin; anti-mouse CD133-PE, anti-mouse CD133-phycoerythrin; anti-mouse ERα, anti-mouse estrogen receptor α; anti-mouse CD24-FITC, anti-mouse CD24-fluorescein isothiocyanate; HRP, horseradish peroxidase; SA-APC, streptavidin-allophycocyanin. Table S3 Frequency of tumors in recipient mice at day 62 after limiting dilution transplantation. ELDA, Extreme Limiting Dilution Analysis; HIF-1α, hypoxia-inducible factor 1α; KO, knockout; MTEC, mammary tumor epithelial cell; TIC, tumor-initiating cell; WT, wild type. Table S4 Frequency of tumors in recipient mice at day 112 after limiting dilution transplantation. ELDA, Extreme Limiting Dilution Analysis; HIF-1α, hypoxia-inducible factor 1α; KO, knockout; MTEC, mammary tumor epithelial cell; TIC, tumor-initiating cell; WT, wild type. Table S5 Summary of the percentage and total number of recipients bearing small tumors at day 112 posttransplant. KO, knockout; WT, wild type. Table S6 Frequency of tumors in recipient mice at day 244 after limiting dilution transplantation. ELDA, Extreme Limiting Dilution Analysis; HIF-1α, hypoxia-inducible factor 1α; KO, knockout; MTEC, mammary tumor epithelial cell; TIC, tumor-initiating cell; WT, wild type. Table S7 Summary of the percentage and total number of recipients bearing small tumors at day 244 posttransplant. KO, knockout; WT, wild type. [file bcr3087-S1.DOCX]

**Additional Figures**

**Figure S1. HIF-1α expression increases during tumor progression in the MMTV-PyMT model and confirmation of HIF-1α deletion.**

**
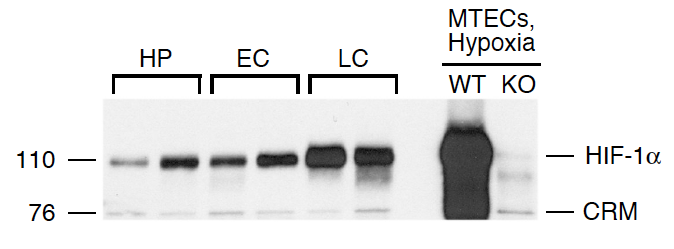
**

**Figure S2. HIF-1α expression increases in response to EGF treatment at normoxia, and EGF prolongs HIF-1α stabilization at hypoxia.**

**
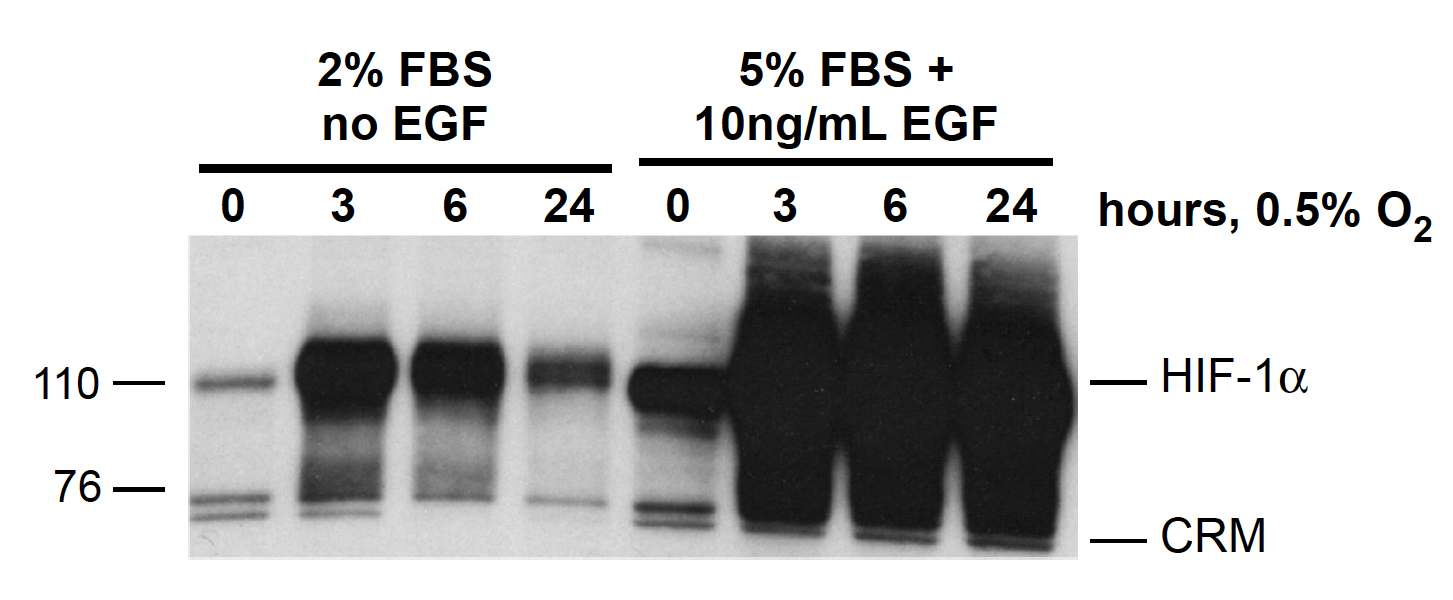
**

**Figure S3. Histology of HIF-1α WT and KO end-stage PyMT tumors.**


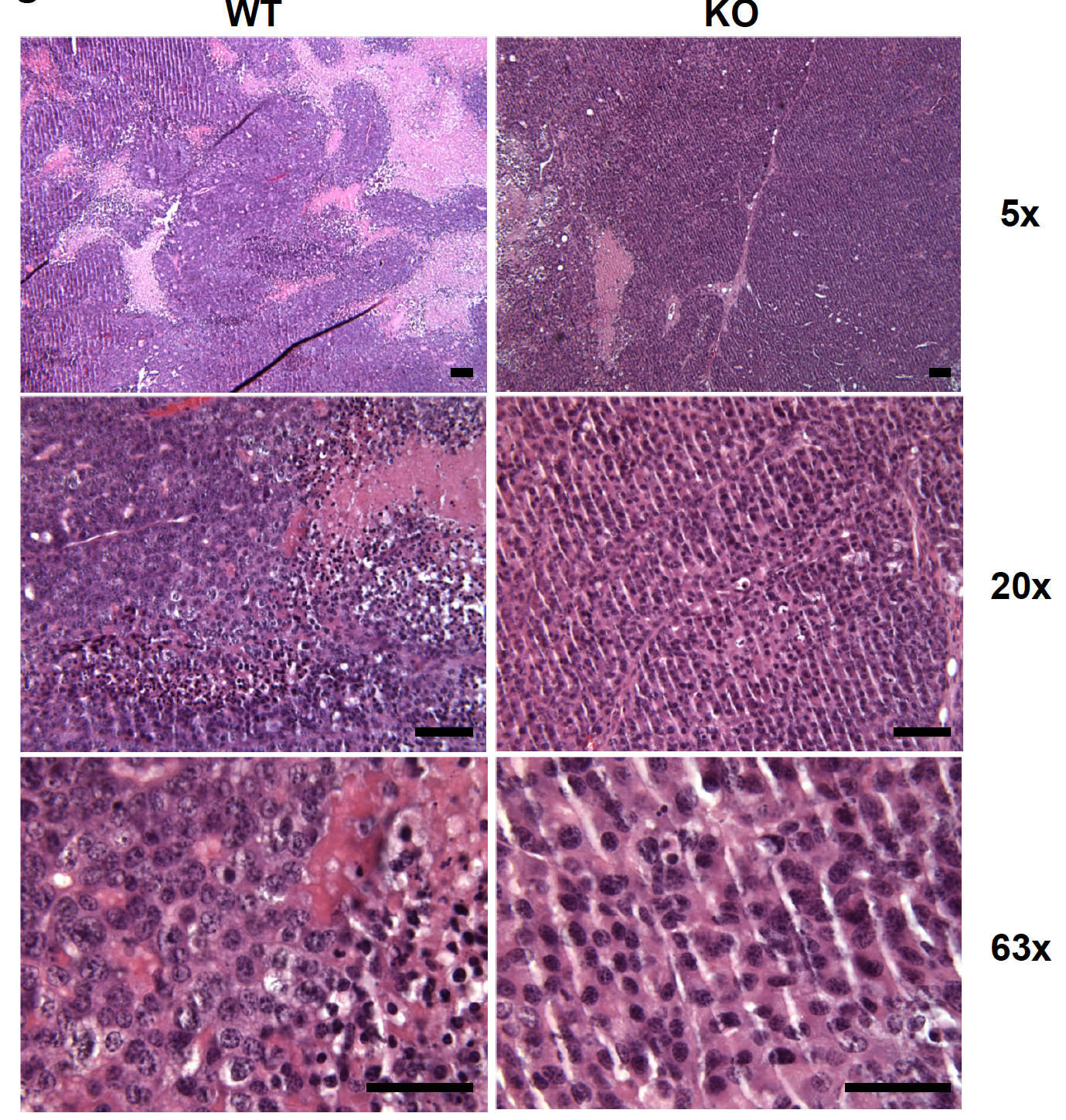


**Figure S4. Ki67 and activated caspase-3 immunostaining in end-stage PyMT tumors.**


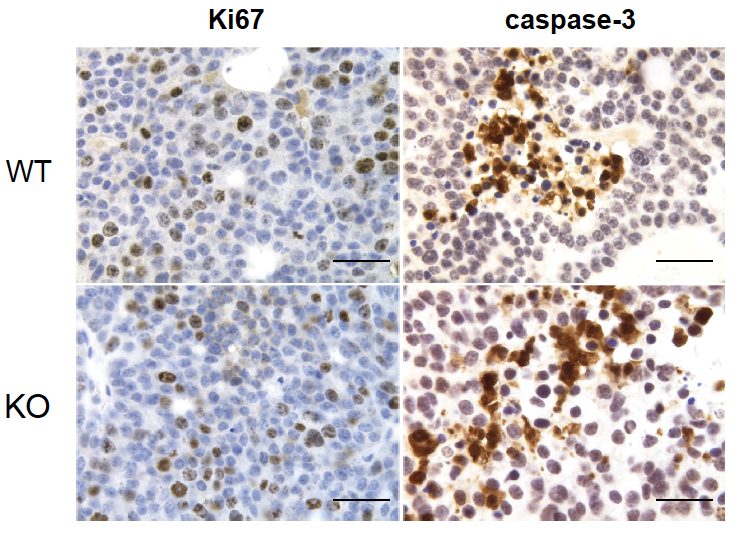


**Figure S5. Expression of p63 and ERα in HIF-1α WT and KO end-stage PyMT tumors.**

**
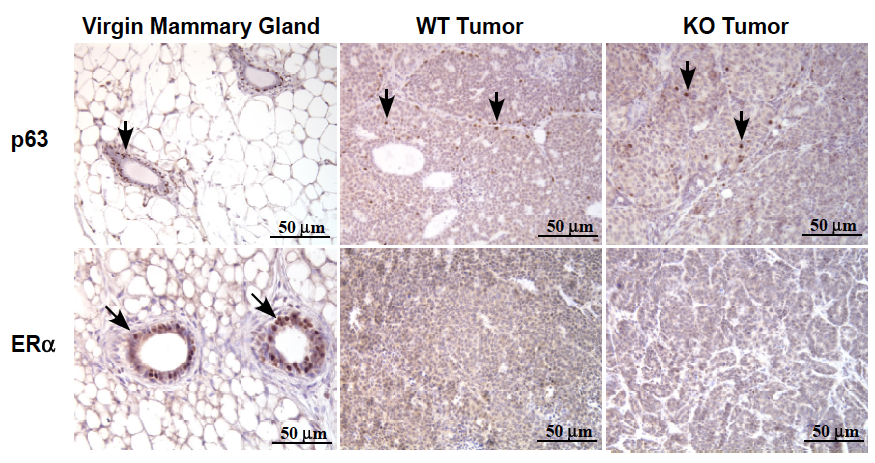
**

**Figure S6. The mean fold-change in sphere formation efficiency (SFE) among biological replicates.**


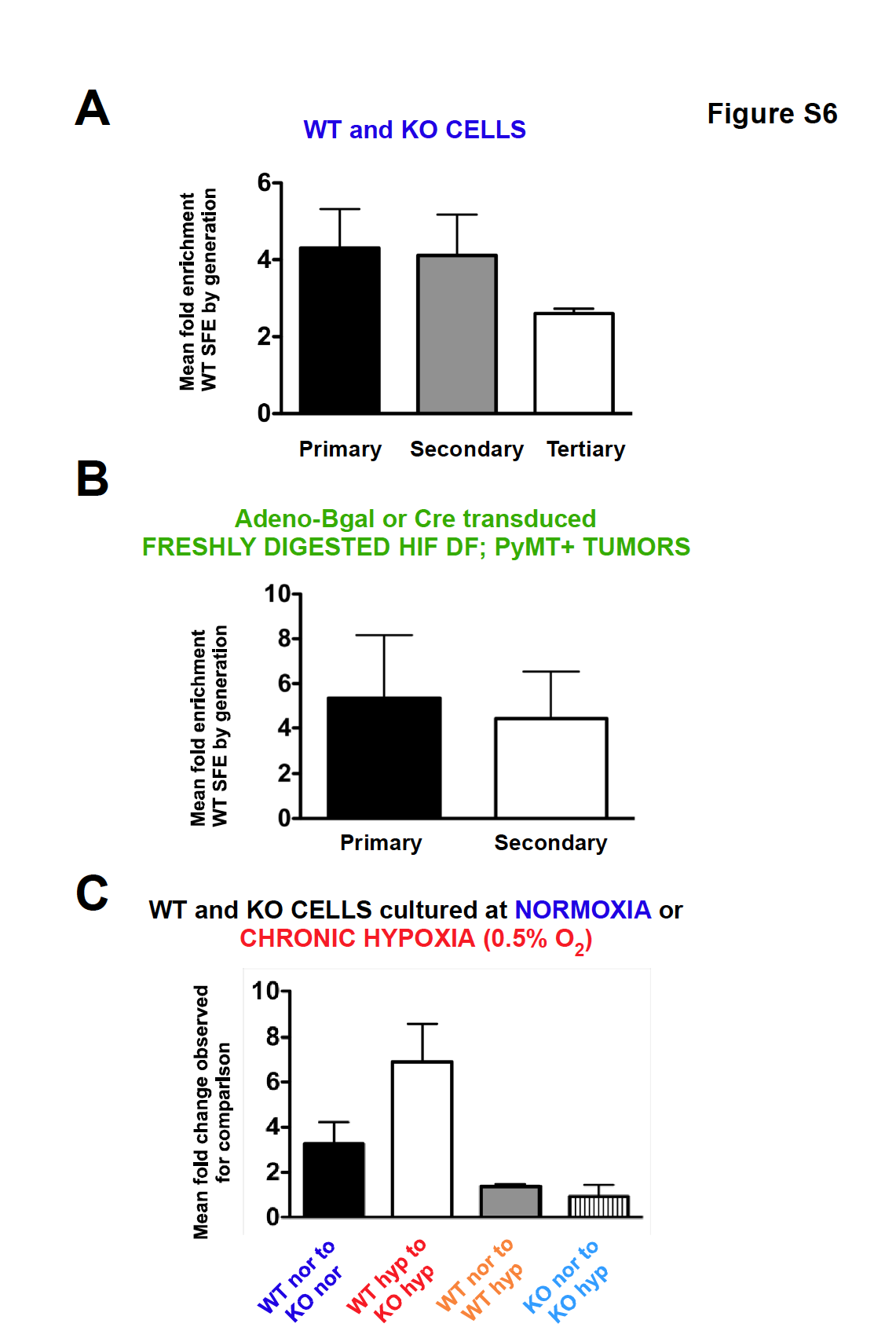


**Figure S7. HIF-1α expression in tumorspheres cultured acutely or chronically at hypoxia.** .


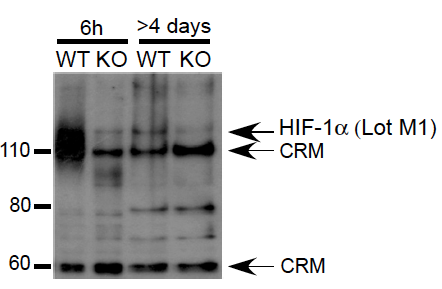


**Figure S8. Gating strategy for sorting CD133^hi^ and CD133^neg^ cells from mammary tumors originating in PyMT+ transgenic femalees.**

**
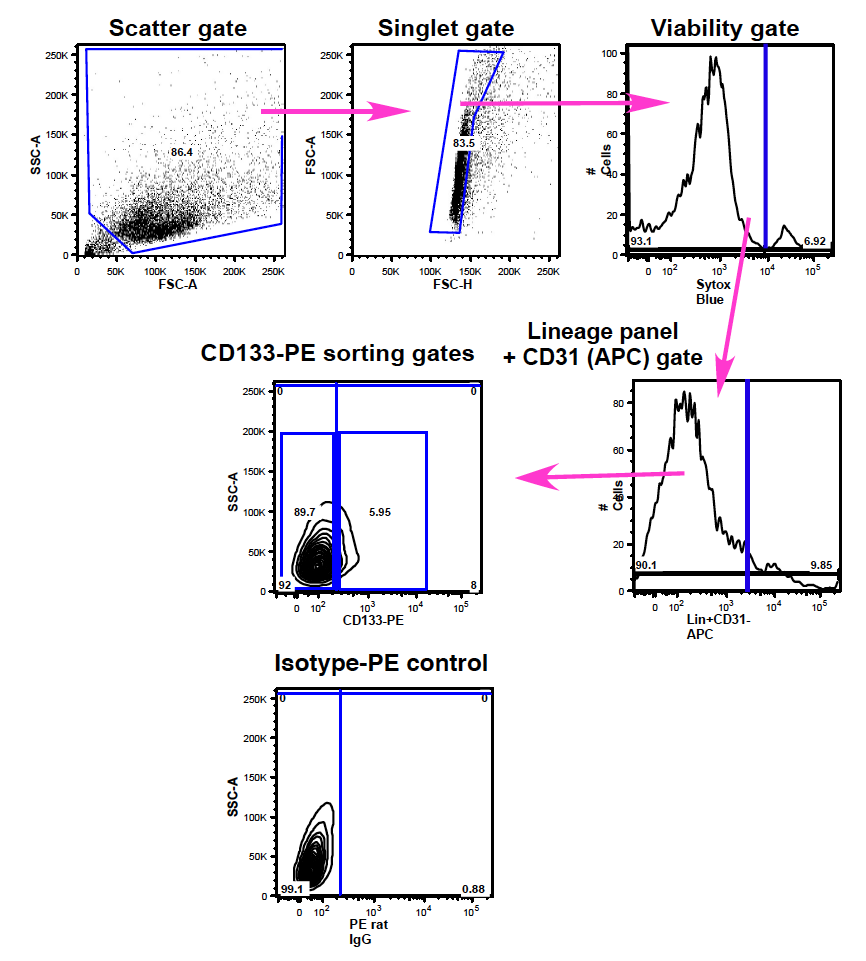
**

**Figure S9. Comparison of WT and KO tumor-initiating potential at day 62 post-transplant.**
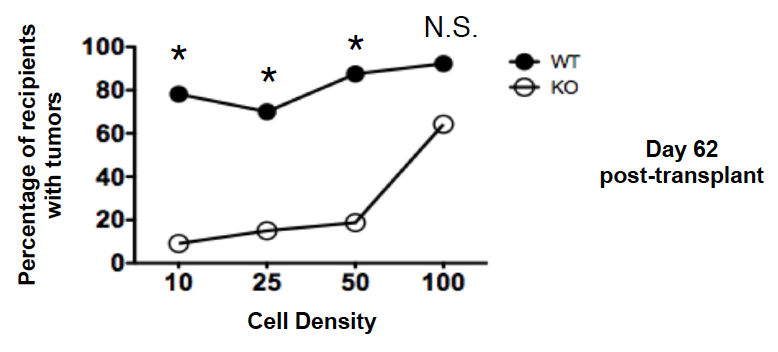


**Figure S10. Mean WT and KO tumor volume in the 200 cell and 500 cell input cohorts up to day 36 post-transplant**.

**
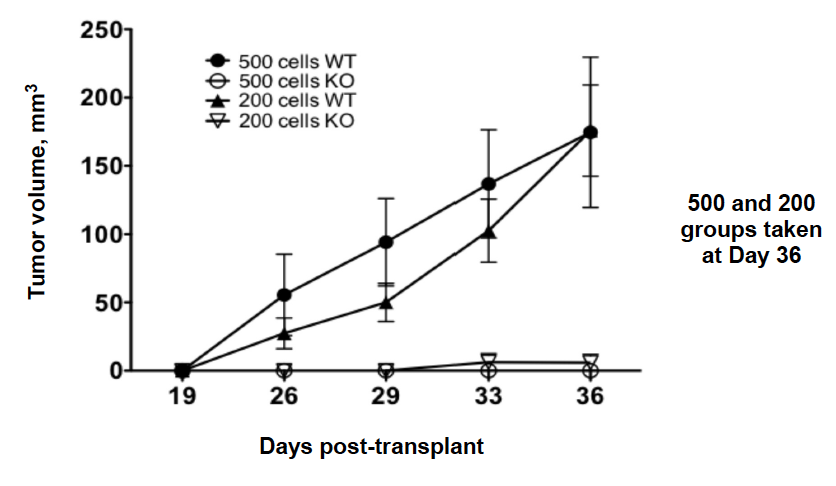
**

**Figure S11. Mean tumor volume in the 100 cell WT and KO cohorts up to day 112 post-transplant.**

**
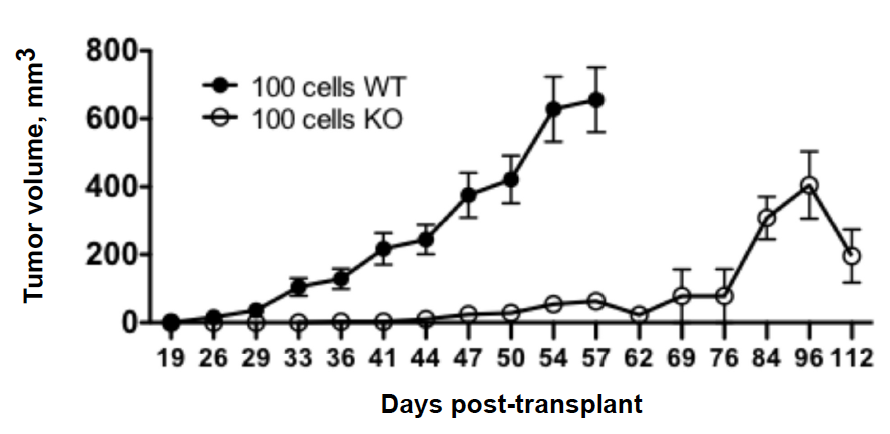
**

**Figure S12. Mean WT and KO tumor volume in the 10, 25 and 50 cell input cohorts up to day 112 post-transplant.**

**
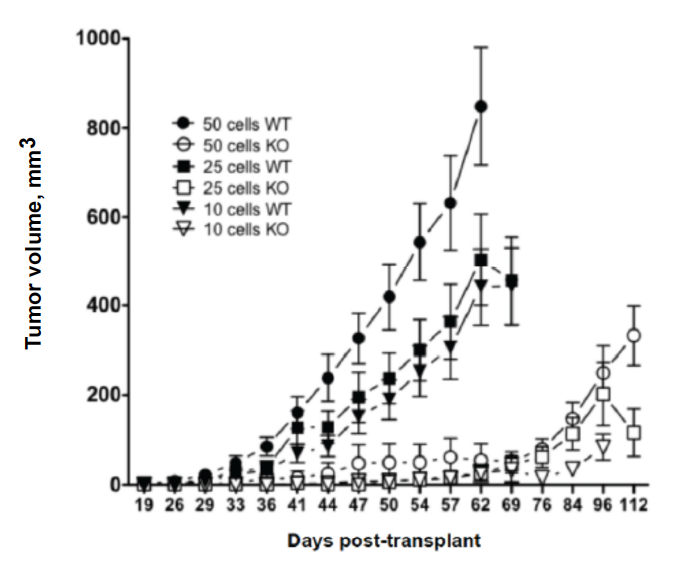
**

**Supplementary Materials and Methods**

**Hyperlink to database to view digitally scanned images of whole slides**

Whole slides were digitally-scanned at high resolution at either 20x or 40x magnification using the Aperio ScanScope system maintained by the Department of Pathology at the UTHSC. A public folder containing the slide images may be accessed by logging into: <http://ap1400-dsr.uthsc.edu/>. The user name is Seagroves and the password is Pathology (each case-sensitive). Once logged into the database, please click “all digital slides”. Individual slides may then be selected for viewing by clicking each file. Clicking the “+” sign will allow the user to digitally zoom into each field of interest at high resolution. Fields of interest are indicated by the highlighted box, which may be moved over any portion of the slide for viewing at a particular magnification. Representative end-stage WT and KO tumors stained with H&E, and WT and KO tumor slides immunostained with p63 antibodies are included in the database.

To view the scanned slides, users must first download the freely available Aperio ImageScope Viewer software (<http://www.aperio.com/download-imagescope-viewer.asp>); a computer running Windows OS is also required.
