# Supplementary material for: Hypoxia-inducible factor 1α promotes primary tumor growth and tumor-initiating cell activity in breast cancer
Source: Breast Cancer Res. 2012 Jan 7;14(1):R6. doi: 10.1186/bcr3087 (PMC3496121; doi:10.1186/bcr3087)
Supplement: Additional file 2 — Figure S1 HIF-1α expression increase during tumor progression in the MMTV-PyMT mouse model and confirmation of HIF-1α deletion. HS-WCE protein extracts were prepared from two individual transgenic MMTV-PyMT female mice (FVB/Nj strain) between 6 and 9 weeks of age. HS-WCE, high-salt whole-cell extract; MMTV-PyMT, mouse mammary tumor virus polyoma virus middle T. By palpation, each mouse had at least one mammary gland with hyperplastic (HP) (palpable as grainy), early-carcinoma (EC) (< 250 mm3) or late-carcinoma (LC) (> 500 mm3) lesions, which was expected because tumor progression among glands is asynchronous in this model. Two independent glands (one from each mouse) per stage were utilized to prepare HS-WCEs for Western blotting. As shown, HIF-1α expression generally increased during progression and was most abundant in late-stage carcinomas. Included as controls were HS-WCEs prepared from wild-type (WT) and knockout (KO) mammary tumor epithelial cells (MTECs) grown to 80% confluence, followed by hypoxia exposure for 6 hours at 0.5% O2. A cross-reactive material (CRM) band at about 76 kDa was detectable when lot E2 of NB 100-479 of the anti-mouse HIF-1α primary antibody (Novus Biologicals, Littleton, CO, USA) was used. KO cells were transduced twice with adenovirus-Cre in monolayer culture at 80 to 100 plaque-forming units/cell to achieve > 99% deletion efficiency as determined by qRT-PCR and Western blot analysis. Figure S2 HIF-1α expression increases in response to EGF treatment at normoxia, and EGF prolongs HIF-1α stabilization at hypoxia. Wild-type (WT) mammary tumor epithelial cells (MTECs) were either cultured in complete growth medium (containing 5% fetal bovine serum, FBS + 5 μg/ml insulin + 10 ng/ml epidermal growth factor, EGF) or in medium supplemented only with 2% FBS for at least five passages prior to replating to test the effect of EGF treatment on HIF-1α expression. Cells grown to 80% confluence were incubated at hypoxia for the number of hours indi [file bcr3087-S2.DOCX]

**Additional Tables**

**Table S1** Primers and Roche Universal Probe Library (UPL) FAM-labeled probes utilized in quantitative real-time PCR assays.

| **Gene** | **Forward Primer** | **Reverse Primer** | **UPL ID** |
| --- | --- | --- | --- |
| *Hif1a* (genomic) | tgagcttgctcatcagttgc | tgagcctcataacagaagctttatc | 60 |
| *Hif1a* (cDNA) | catgatggctccctttttca | gtcacctggttgctgcaata | 98 |
| *Vegf* | aacgatgaagccctgagt | aggtttgatccgcatgatct | 9 |
| *Pgk1* | tacctgctggctggatgg | cacagcctcggcatatttct | 108 |
| *Slc2a1* (Glut-1) | atggatcccagcagcaag | ccagtgttatagccgaactgc | 52 |
| *Prom1* (CD133) | gaaggagcccagcttagagg | ggtcattcactcaaagtaccatcc | 19 |
| *Notch1* | actatctcggcggcttttc | ggcactcgttgatctcctct | 5 |
| *Notch2* | tgcctgtttgacaactttgagt | gtggtctgcacagtatttgtcat | 6 |
| *Notch3* | agctgggtcctgaggtgat | agacagagccggttgtcaat | 9 |
| *Notch4* | ggacctgcttgcaaccttc | ctcacagagcctcccttcc | 34 |
| *Hey1* | catgaagagagctcacccaga | cgccgaactcaagtttcc | 17 |
| *Hey2* | gtggggagcgagaacaatta | gttgtcggtgaattggacct | 104 |
| *Hes1* | tgccagctgatataatggagaa | ccatgataggctttgatgacttt | 20 |
| *Hes2* | agctgcgcaagaacctaaag | aacttcgaagagcgggaagt | 3 |
| *Dll1* | gggacagaggggagaagatg | cacaccctggcagacagat | 20 |
| *Jagged1* | gaggcgtcctctgaaaaaca | acccaagccactgttaagaca | 6 |
| *Jagged2* | tctgtgaggacctggtggat | ggttcacagagatccatgtcc | 26 |
| *Snail1* | gtctgcacgacctgtggaa | caggagaatggcttctcacc | 71 |
| *Slug (Snail2)* | tgcaagatctgtggcaagg | cagtgagggcaagagaaagg | 71 |
| *Twist* | agctacgccttctccgtct | tccttctctggaaacaatgaca | 58 |
| *Fibronectin (Fn1)* | cggagagagtgcccctacta | cgatattggtgaatcgcaga | 52 |
| *Ints3* | gtggctgttattgactctgcac | caggttccccatcatcacat | 17 |
| *cytokeratin 18* | agatgacaccaacatcacaagg | cttccagaccttggacttcct | 78 |

All assays were designed using the Roche Universal ProbeLibrary Assay Design Center (<http://qpcr.probefinder.com/organism.jsp>).

**Table S2** Primary antibody source and dilution factors utilized in western blotting, immuno-histochemistry (IHC), immunofluorescence (IF) and FACS.

| **Antibody** | **Source (Catalog #)** | **Dilution** | **Purpose** | **Secondary** |
| --- | --- | --- | --- | --- |
| anti-mouse HIF-1α | Novus Biologicals (NB100-479, Lots E2 or M1) | 1:5,000 | western | 1:50,000 anti-rabbit HRP |
| anti-mouse Lamin A/C | Santa Cruz Biotechnology (sc-6215) | 1:1,000 | western | 1:50,000 anti-goat HRP |
| anti-mouse−SMA | Sigma-Aldrich (A5228) | 1:5,000 | western | 1:50,000 anti-mouse HRP |
| anti-mouse Ki67 | Santa Cruz Biotechnology (sc-7846) | 1:500 | IHC | 1:200; Vector Elite kit |
| anti-mouse caspase3, active | R&D Systems (AF835) | 1:750 | IHC | 1:200; Vector Elite kit |
| anti-mouse CD133-PE | eBiosciences (clone AC133) | 1:100  1:200 | FACS  IF | N/A |
| anti-mouse CD133 | Millipore (clone 13A4) | 1:50 | IF | 1:500 AlexaFluor488 or 594 |
| anti-mouse ERα | Santa Cruz Biotechnology (sc-542) | 1:10,000 | IHC | 1:200; Vector Elite kit |
| anti-mouse p63 | Abcam (ab53039) | 1:20,000 | IHC | 1:200; Vector Elite kit |
| anti-mouse keratin 14 | Covance (clone AF64) | 1:750 | IF | 1:500 AlexaFluor594 |
| anti-mouse keratin 5 | Abcam (ab52635) | 1:100 | IF | 1:500 AlexaFluor594 |
| anti-mouse keratin 8 | DSHB (Troma-1) | 1:20 | IF | 1:500 AlexaFluor488 |
| anti-mouse CD24-FITC | BD Biosciences (553261) | 1:100 | FACS | N/A |
| anti-mouse linage panel-biotin conjugated | BD Pharmingen (559971) | 1:100 | FACS | SA-APC  (BD Pharmingen 554067) |
| anti-mouse CD31-biotin conjugate | BD Pharmingen (553371) | 1:100 | FACS | SA-APC |

**Table S3** Frequency of tumors in recipient mice at day 62 after limiting dilution transplantation.

_________________________________________________________________________________________

**Day 62 Post-Transplant**

**Genotype of MTECs**

**Number** **HIF-1α WT** **HIF-1α KO**

**of cells**

**injected** **Tumor-positive** **Tumor-positive** **Fisher’s exact test**

100 92% (12/13) 50% (7/14) N.S.

50 88% (14/16) 19% (3/16) *p*= 0.0002

25 70% (14/20) 15% (3/20) *p*= 0.0011

10 78% (18/23) 9% (2/22) *p<* 0.0001

**Estimated HIF-1α WT** **HIF-1α KO Chi-square test**

**TIC freq.**

**by ELDA** 1/18 1/135 *p*= 3.96e^-^14

**(95% C.I.)** (1/13-1/26) (1/84-1/217)

_________________________________________________________________________________________

A Fisher’s exact (Chi-square) test was utilized to compare significance of tumor-initiating potential between WT and KO cells at each cell density (N.S., not significant). The estimated TIC frequency at day 62 post-transplant was determined by ELDA software.

**Table S4** Frequency of tumors in recipient mice at day 112 after limiting dilution transplantation.

_________________________________________________________________________________________

**Day 112 post-transplant**

**Gen****otype of MTECs**

**Number** **HIF-1α WT** **HIF-1α KO**

**of cells**

**injected** **Tumor-positive** **Tumor-positive** **Fisher’s exact test ***

100 100% (13/13) 100% (14/14) test not appropriate

50 94% (15/16) 94% (15/16) N.S.

25 95% (19/20) 50% (10/20) *p*= 0.0011

10 96% (22/23) 45% (10/22) *p<* 0.0001

* ELDA analysis could not estimate TIC frequency with a 95% CI based on these data

_________________________________________________________________________________________

A Fisher’s exact (Chi-square) test was utilized to compare TIC potential between WT to KO cells at each cell density. Data for the 10 and 25 cell input groups remain statistically significant, whereas no significant differences (N.S.) were observed between WT and KO cells for the 50 cell input groups. The Chi-square test is no longer appropriate for the 100 cell input group, as all recipient mice bearing WT or KO tumor cells had developed measurable tumors by day 112.

**Table S5** Summary of the percentage and total number of recipients bearing small tumors at day 112 post-transplant.

| **Genotype-**  **Cell density** | **% tumor-positive recipients with tumor <250mm^3^** |
| --- | --- |
| WT- 100 | 0% (0/13) |
| KO- 100 | 7% (1/14) |
| WT- 50 | 0% (0/15) |
| KO- 50 | 13.3% (2/15) |
| WT- 25 | 5.2% (1/19) |
| KO- 25 | 20% (2/10) |
| WT- 10 | 9% (2/22) |
| KO- 10 | 60% (6/10) |

**Table S6** Frequency of tumors in recipient mice at day 244 after limiting dilution transplantation.

_________________________________________________________________________

**Day 244 Post-Transplant**

**Genotype of MTECs**

**Number** **HIF-1α WT** **HIF-1α KO**

**of cells**

**injected** **Tumor-positive** **Tumor-positive** **Fisher’s exact test ***

50 100% (16/16) 100% (16/16) test not appropriate

25 100% (20/20) 95% (19/20) N.S.

10 96% (22/23) 73% (16/22) *p<* 0.047

* ELDA analysis could not estimate TIC frequency with a 95% CI based on these data

______________________________________________________________________

A Fisher’s exact test was utilized to compare significance of tumor-initiating potential between WT to KO cells for the 10 and 25 cell input groups. The Chi-square test is no longer appropriate for the 50 cell input group, as all recipient mice bearing WT or KO tumor cells had developed measurable tumors by day 244.

| **Genotype-Cell density** | **% of tumor-positive recipients with tumors**  **< 250mm^3^** |
| --- | --- |
| WT- 50 | 0% (0/16) |
| KO- 50 | 0% (0/16) |
| WT- 25 | 0% (0/20) |
| KO- 25 | 10.5% (2/19) |
| WT- 10 | 0% (0/22) |
| KO- 10 | 37.5% (6/16) |

**Table S7** Summary of the percentage and total number of recipients bearing small tumors at day 244 post-transplant.
